# Expression of melanoma differentiation–associated gene 5 in the epidermis and cutaneous deposition of complement C3 and immunoglobulins in patients with dermatomyositis

**DOI:** 10.1371/journal.pone.0351248

**Published:** 2026-06-08

**Authors:** Yoshiaki Zaizen, Takuma Koga, Masahiro Tsutsumi, Shinjiro Kaieda, Jun Akiba, Takekuni Nakama, Tomoaki Hoshino

**Affiliations:** 1 Division of Respirology, Neurology and Rheumatology, Department of Medicine, Kurume University School of Medicine, Fukuoka, Japan; 2 Department of Pathology Informatics, Nagasaki University Graduate School of Biomedical Sciences, Nagasaki, Japan; 3 Department of Dermatology, Kurume University School of Medicine, Fukuoka, Japan; 4 Department of Pathology, Kurume University School of Medicine, Fukuoka, Japan; Kwame Nkrumah University of Science and Technology, GHANA

## Abstract

**Objectives:**

Dermatomyositis (DM) is an autoimmune disease characterized by interface dermatitis, but the immunopathological features underlying cutaneous inflammation remain incompletely understood. The aim of this study was to characterize the cutaneous deposition of complement and immunoglobulins, as well as to clarify the localization of melanoma differentiation-associated gene 5 (MDA5) in DM skin.

**Methods:**

Skin biopsy specimens from 22 patients with DM and 13 control specimens obtained from cancer-free skin of patients with dermatofibrosarcoma protuberans were examined. Immunohistochemical staining for complement C3c, immunoglobulins (IgG, IgM, and IgA), and MDA5 was semi-quantitatively evaluated, focusing on the superficial dermis near the dermo-epidermal junction.

**Results:**

Significantly greater deposition of C3c, IgM, and IgA was exhibited by DM skin compared with control skin (all p ≤ 0.001), predominantly localised to the superficial dermis at sites of interface dermatitis. In contrast, IgG showed comparable deposition in both DM and control skin. MDA5 was strongly expressed in the stratum spinosum and basal layer of the epidermis in both DM and control skin. Enhanced MDA5 expression was notably observed in dermal inflammatory cells and capillaries in DM skin, but minimal expression was observed in the dermis of control skin.

**Conclusions:**

DM skin is characterized by the deposition of immunoglobulins and complement C3c at sites of interface dermatitis, findings that are consistent with immune complex-mediated injury. MDA5 is widely expressed in both DM and control skin epidermis and can be detected in the infiltrating inflammatory cells of DM.

## Introduction

Dermatomyositis (DM) is a rare autoimmune disease characterised by dermatitis and skeletal muscle weakness concurrent with muscle inflammation [1]. The major cutaneous manifestations include Gottron’s sign, the heliotrope rash, mechanic’s hands, and the shawl sign [2,3]. Several organs are postulated to be involved in DM, with pulmonary involvement considered the most common [4]. Interstitial lung disease (ILD) is a major complication of DM. Some patients with DM experience rapidly progressive ILD with poor outcomes [5], particularly those positive for autoantibodies against melanoma differentiation-associated gene 5 (MDA5) [6]. We have previously reported that several variants in the interferon-induced with helicase C domain 1 gene, which encodes MDA5, are associated with an increased risk of rapidly progressive ILD in patients with DM [7].

The pathogenesis of muscle tissue involvement in patients with DM has been described in several reports [8,9]. In the muscle tissue of patients with DM, capillary destruction and vascular endothelial injury can lead to luminal obliteration, necrosis, and thrombosis [8,9]. In addition, Kissel et al. have demonstrated the deposition of the C5b-9 membranolytic attack complex in the microvasculature in DM [10], suggesting the involvement of complement components in vascular injury in DM. We previously reported strong expression of MDA5 protein in the lungs of patients with DM-associated ILD (DM-ILD). Furthermore, we demonstrated prominent deposition of complement component C3c and immunoglobulins (IgG, IgM, and IgA) in DM-ILD lung tissue [11]. These findings suggest that the high expression of MDA5 protein and immune complexes may contribute to tissue injury in DM. However, the mechanisms underlying skin involvement remain unclear.

Herein, we conducted an exploratory study to examine the deposition of immunoglobulins (IgG, IgM, IgA) and complement component C3 in DM and control skin. Additionally, we evaluated the expression of MDA5 protein using an in-house monoclonal antibody (clone H27) [11]. Based on these observations, we subsequently included additional cases and conducted an expanded cohort study.

## Materials and methods

### Ethical considerations

This single-centre retrospective study consisted of two parts: an exploratory study and an expanded cohort study. Both the exploratory and expanded cohort studies were conducted in accordance with the tenets of the Declaration of Helsinki and approved by the Ethics Board of Kurume University (approval date, July 31, 2019, No. 19090; and approval date, May 27, 2025, No. 25072, respectively). In the exploratory study, informed consent was obtained from all patients via the opt-out method. In the expanded cohort study, written informed consent was obtained for most patients; however, for patients who did not visit our institute during the study period, informed consent was obtained via the opt-out approach.

For the exploratory study, case extraction and clinical information collection were conducted from May 8 to June 3, 2020. The immunohistochemical evaluation was completed by September 30, 2020. For the expanded cohort study, case selection and clinical information collection were conducted from June 17 to July 8, 2025, and the immunohistochemical evaluation was completed by September 26, 2025.

### Study subjects

In the exploratory study, we investigated the skin biopsy samples from six consecutive patients who were diagnosed with DM at our institute between 2019 and 2022. The patients met the diagnostic criteria for polymyositis and DM as reported by Bohan and Peter [12,13], or the diagnostic criteria for clinically amyopathic DM [14]. We examined skin specimens from patients with DM that exhibited obvious dermatitis. None of the patients with dermatomyositis had received systemic corticosteroids or immunosuppressive agents at the time of skin biopsy. As controls, we examined the skin samples of cancer-free areas from six patients with dermatofibrosarcoma protuberans (DFSP) who underwent skin resection at Kurume University Hospital (Kurume, Fukuoka, Japan) between 2019 and 2022. We selected cases of DFSP as controls because, among cutaneous malignant tumors, DFSP typically requires the widest surgical margins. At our institution, DFSP is routinely excised with a 4-cm surgical margin. This allows for an evaluation of the resection margins with minimal influence from tumor-associated inflammation.

In the expanded cohort study, we investigated skin samples from 16 patients with DM and seven patients with DFSP, who served as controls, following the same methodology as in the exploratory study. The expanded cohort study period ranged from 2015 to 2024. All cases, except those enrolled in the exploratory study, were included in the expanded cohort study.

The expanded cohort was assembled to confirm the observations obtained in the exploratory study using the same immunohistochemical and statistical methods. As originally planned, the final analysis was performed using the pooled dataset after confirming that the findings were directionally consistent between the two cohorts.

### Establishment of an anti-human MDA5 Monoclonal Antibody (mAb)

We established an in-house anti-human MDA5 mAb, as reported previously [11,15]. Briefly, full-length human MDA5 cDNA (GenBank accession no. AF095844) with a 6X His tag, GST, and a turbo 3C protease cleavage site (Leu-Glu-Val-Leu-Phe-Gln-Gly-Pro) at the N-terminus was subcloned into the pPSC8 expression vector (Protein Sciences Corporation, Meriden, CT, USA), hereafter referred to as pPSC8/human MDA5. Recombinant human MDA5 protein was isolated from SF9 cells co-transfected with baculovirus AcNPV and pPSC8/human MDA5. An anti-human MDA5 mAb clone H27 (mouse IgG1) was established by fusing the mouse myeloma cell line X-63·Ag8/653 with spleen cells isolated from a BALB/c mouse immunised with the recombinant human MDA5 protein. Purified antibody was isolated from the antisera using a protein G column (Cytiva, Tokyo, Japan), as reported previously [16,17]. The specificity and utility of clone H27 for detecting human MDA5 protein have been demonstrated in our previous studies using lung tissue and human serum samples [11,15]. In situ analysis revealed that H27 detects human MDA5 protein in regions where human MDA5 mRNA is strongly expressed. In addition, H27 detects human MDA5 protein but not mouse MDA5 protein.

### Immunohistochemical staining

Direct immunofluorescence using frozen tissue sections is the standard method for evaluating immunoglobulin and complement deposition in dermatopathology. In the present study, we used formalin-fixed paraffin-embedded archival specimens because frozen tissue was not available for this retrospective analysis and all specimens had been collected as part of routine clinical practice.

We performed immunohistochemical staining using the aforementioned in-house anti-human MDA5 mAb (clone H27), anti-human complement C3c antibody, and anti-human immunoglobulin (IgG, IgA, IgM) antibodies as previously described [11,18]. Briefly, skin tissues were fixed with 10% buffered formalin and embedded in paraffin wax. Serial sections (4-μm-thick) were cut from the paraffin-embedded tissues and placed on poly-L-lysine-coated slides. Deparaffinised sections were autoclaved for 3 min in 10 mM citric acid buffer (pH 6.0). The sections were incubated in 0.3% H_2_O_2_ for 10 min to block endogenous peroxidase activity. Subsequently, they were stained using mouse anti-human MDA5 mAb (H27; 1 μg/mL), rabbit anti-human IgG (P0214; Agilent, Palo Alto, CA, US; x100), rabbit anti-human IgM (P0215; Agilent; x50), rabbit anti-human IgA (P0216; Agilent; x100), and rabbit anti-human complement C3c (A0062, Agilent; x5000); mouse IgG1 (BioLegend Japan, Tokyo, Japan) was used as a negative control for the in-house mouse anti-human MDA5 monoclonal antibody (clone H27, mouse IgG1). Separate species-matched negative controls were not performed for the rabbit primary antibodies against C3c and immunoglobulins. The sections were treated with these antibodies at 20℃ for 2 h. Positive reactivity was identified using goat anti-mouse and anti-rabbit immunoglobulins conjugated to a peroxidase-labelled polymer (EnVision Dual Link system-HRP, Agilent) and Liquid DAB + Substrate Chromogen System Liquid (Agilent). Additionally, haematoxylin & eosin (HE) staining was performed using deparaffinised sections.

### Pathological assessment and statistical analysis

We assessed the immunohistochemistry staining intensity in the superficial dermis near the dermo-epidermal junction using a previously reported methodology [11]. Slides were rated as follows: score 0, negative; score 1, weakly positive; score 2, moderately positive; and score 3, strongly positive. All pathological evaluators performed semi-quantitative scoring while blinded to the clinical information. Three evaluators (YZ, MT, and JA) independently reviewed all slides while blinded to clinical information, and the final scores were determined by consensus.

We performed statistical analysis for these semi-quantitative scores using the Mann–Whitney U test. Because five immunohistochemical markers were compared between groups, Bonferroni correction was additionally performed as a sensitivity analysis. A p value of <0.01 was considered statistically significant after correction. All statistical analyses were performed using JMP 18.0 (SAS Institute, Cary, NC, US).

## Results

### Patient characteristics

Table 1 presents patient background characteristics. In this study, skin tissues were obtained from 22 patients with DM; skin samples from 13 patients with DFSP served as the control group. Seven patients with DM were positive for anti-MDA5 autoantibodies, while fifteen patients exhibited a positive response for anti-transcription intermediary factor 1 gamma (TIF1-γ) autoantibodies. In the present study, all patients with DM who underwent skin biopsy during the study period were included; however, no patients with dermatomyositis positive for anti–aminoacyl–transfer RNA synthetase antibodies or anti–Mi-2 antibodies were identified.

**Table 1:** Patient characteristics.

	Total	Exploratory study	Expanded cohort study
Number	35	12	23
Age	50 (39-68)	47 (29-60)	54 (40-71)
Sex: Female	14 (40%)	7 (58%)	14 (61%)
Diagnosis			
DM/CADM*	22 (63%)	6 (50%)	16 (70%)
DFSP (control)	13 (37%)	6 (50%)	7 (30%)
Positive antibody in DM			
Anti-MDA5	7 (32%)	2 (33%)	5 (31%)
Anti-TIF1-γ	15 (68%)	4 (67%)	11 (69%)
Biopsy site			
Head	2 (6%)	1 (8%)	1 (4%)
Trunk	18 (51%)	7 (58%)	11 (48%)
Upper limb	12 (34%)	2 (17%)	10 (43%)
Lower limb	3 (9%)	2 (17%)	1 (4%)

Abbreviations: CADM, clinically amyopathic dermatomyositis; DM, dermatomyositis; DFSP, Dermatofibrosarcoma protuberans; MDA5, melanoma differentiation-associated gene 5; TIF1, transcription intermediary factor 1

* Four patients had clinically amyopathic dermatomyositis (one in the exploratory study and three in the expanded cohort study).

### Both DM and control skin showed strong epidermal expression of MDA5 protein

**Fig 1:**
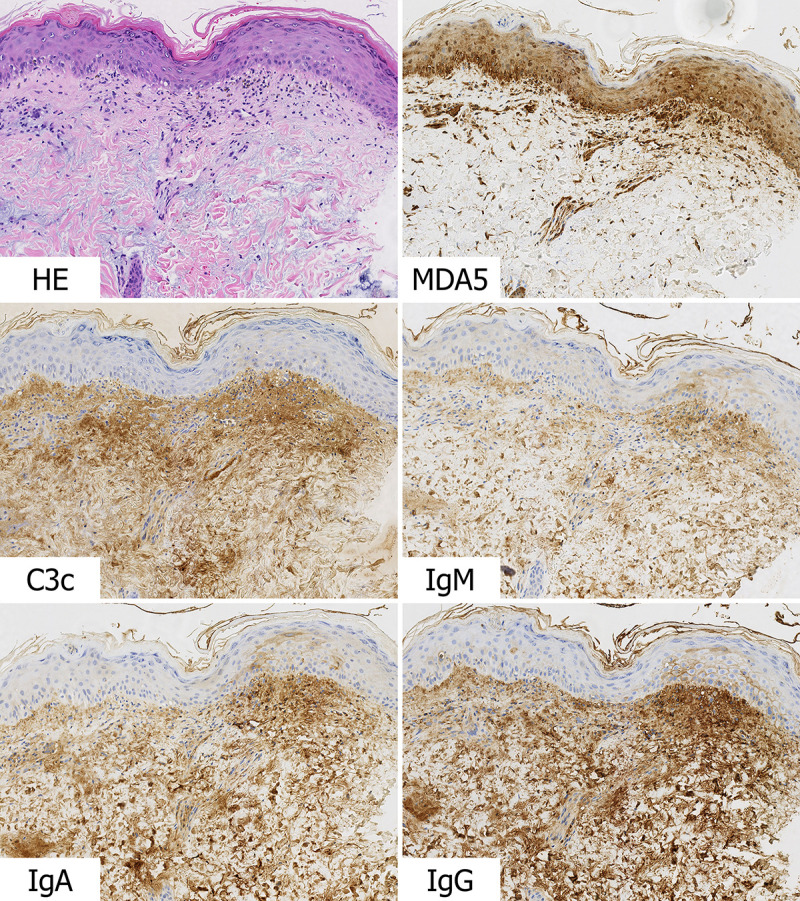
Haematoxylin & eosin staining and immunohistochemical staining images of skin samples obtained from a patient with dermatomyositis. Patients with dermatomyositis exhibit lymphocyte-predominant inflammatory cell infiltration at the epidermal-dermal junction and in the superficial dermis, presenting with interface dermatitis. MDA5 is expressed in the spinous and basal layers of the epidermis, while complement and immunoglobulins are strongly deposited in the superficial dermis near the epidermal-dermal junction.

**Fig 2:**
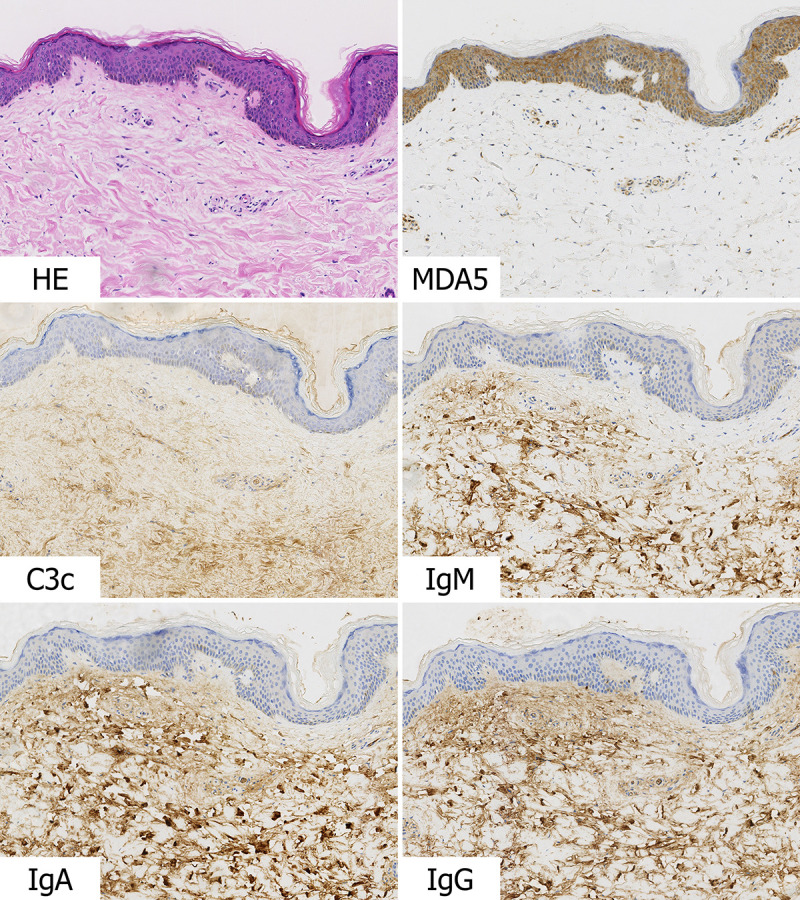
Haematoxylin & eosin staining and immunohistochemical staining images of control skin samples obtained from a patient with dermatofibrosarcoma protuberans who underwent skin resection. MDA5 expression is observed in the epidermis of control subjects; however, the deposition of complement components and immunoglobulins in the dermis is weaker than that in patients with dermatomyositis.

Fig 1 shows representative images (HE staining, C3c, IgM, IgG, IgA, and MDA5) from a patient with DM who was positive for anti-MDA5 autoantibody. Fig 2 shows corresponding representative images from control skin. HE staining demonstrated inflammatory infiltrates in the superficial dermis near the dermo-epidermal junction in all DM samples. Interface dermatitis, characterised by basal cell vacuolisation and apoptotic keratinocytes, was consistently observed in DM skin (left upper panel in Fig 1). Immunohistochemical staining revealed high expression of MDA5 in the stratum spinosum and basal layer of the epidermis in both DM and control skin. In DM skin, MDA5 immunoreactivity was also observed in structures morphologically consistent with dermal capillaries and infiltrating inflammatory cells, whereas such staining was minimal in the dermis of control skin (right upper panel in Figs 1 and 2). These findings reflect differences in cellular localisation associated with inflammatory cell infiltration rather than increased epidermal MDA5 expression in DM.

Table 2 presents the semi-quantitative immunostaining scores of this study. MDA5 staining was strong in both the DM and control groups. In the DM group, strong MDA5 expression was consistently observed in all cases, regardless of positivity for anti-MDA5 antibodies. In this cohort, MDA5 expression was relatively weak in six cases. However, these were older specimens that had been stored for extended periods, which may have affected the staining intensity.

**Table 2:** Statistical analysis of the results of immunohistochemical staining.

Expression intensity 0/1/2/3 Median (IQR)	DM (n = 22)	Control (n = 13)	p-value
C3c	0/3/7/12 3 (2-3)	5/4/3/1 1 (0-2)	0.0003*
IgM	2/6/9/5 2 (1-2)	7/4/2/0 0 (0-1)	0.0012*
IgG	0/8/6/8 2 (1-3)	2/2/7/2 2 (1-2)	0.4292
IgA	0/4/5/13 3 (2-3)	3/5/4/1 1 (1-2)	0.0011*
MDA5	0/2/5/15 3 (2-3)	0/4/4/5 2 (1-3)	0.0628

* Statistically significant.

Abbreviations: IQR, interquartile range; DM, dermatomyositis; Ig, immunoglobulin; MDA5, melanoma differentiation-associated gene 5.

Expression intensity score 0, negative; score 1, weakly positive; score 2, moderately positive; and score 3, strongly positive. Details are described in the methods section.

The results of the exploratory study and the expanded cohort study analyzed separately are provided in S1 Table. Overall staining patterns and group differences were generally consistent between the two cohorts. In the expanded cohort, MDA5 staining scores were significantly lower in control skin than in dermatomyositis skin; however, several control specimens were older archival samples, and reduced staining intensity related to long-term storage may have contributed to this finding.

### DM skin exhibited higher C3c and immunoglobulin deposition than control skin

Complement components and immunoglobulins showed strong deposition in the superficial dermis near the dermo-epidermal junction in DM skin, where strong inflammation was observed (Fig 1). In the control skin, these were weakly stained in the deep and superficial dermis (Fig 2).

The semi-quantitative immunostaining scoring results for all cases are shown in Table 2. The median intensity scores of C3c were 3 (interquartile range [IQR], 2–3) in DM skin and 1 (IQR, 0–2) in control skin. The median intensity scores of IgM were 2 (IQR, 1–2) and 0 (IQR, 0–1), respectively, and those of IgA were 3 (IQR, 2–3) and 1 (IQR, 1–2), respectively. We observed significantly greater deposition of C3c, IgM, and IgA in DM skin than in control skin. In contrast, IgG showed moderate to strong deposition in both DM and control skin, with no significant differences between the two groups (p = 0.4292).

## Discussion

Previous studies have reported vascular endothelium injury and the deposition of immune complexes containing C3bNEO and the C5b-C9 membrane attack complex in DM skin [10,19,20]. Additionally, IgG and IgA production by epidermal cells has been reported, with these immunoglobulins frequently observed in epidermal cells and the epidermal stratum basale in several autoimmune diseases, as well as in control skin [21,22]. Herein, we demonstrated that C3c, IgM, and IgA exhibited significantly greater deposition in the skin obtained from patients with DM than in control skin, particularly at the dermo-epidermal junction and in the superficial layers of the dermis, where inflammation was the most intense. Herein, IgG showed moderate or strong deposition in both DM and control skin, corroborating previous findings [21,22]. Our results suggest that C3 and multiple immunoglobulins are involved in the development of interface dermatitis in DM. These findings are consistent with a possible role of immune complex-mediated injury in the development of dermatitis in DM. Taken together, these findings suggest that skin lesions in dermatomyositis are best characterized as interface dermatitis accompanied by immunoglobulin and complement deposition, findings that are consistent with immune complex-mediated injury rather than nonspecific immunoglobulin accumulation.

In our previous report, complement components and immunoglobulins showed strong deposition in a mouse model of lung injury induced by the administration of an anti-human MDA5 polyclonal antibody to human MDA5 transgenic mice [11]. Funabiki et al. reported that a mutant mouse model harbouring a single missense mutation in MDA5 (G821S) spontaneously developed lupus-like nephritis and systemic autoimmune symptoms. These mice presented with chronic inflammation and nephritis, exhibiting immunoglobulin and complement deposition, upregulation of inflammatory cytokines and chemokines in the kidneys, increased immunoglobulin deposition, and positivity for antinuclear and anti-DNA antibodies in the serum [23]. In a review article [24] on the pathogenesis of anti-MDA5 antibody-positive DM, Lu et al. [24] suggested that in the skin lesions in anti-MDA5 antibody-positive DM, keratinocyte-derived interferon stimulates macrophages to produce high levels of interferon gamma-induced protein 10 (IP-10, also known as C-X-C motif chemokine ligand 10; CXCL10), leading to the recruitment of T cells and the induction of dermatitis. Herein, we showed immune complex deposition and severe infiltration of inflammatory cells in DM skin but not in control skin. Collectively, these findings suggest that immunoglobulin and complement deposition, together with inflammatory cell infiltration, may contribute to cutaneous inflammation in patients with DM. Further analysis is needed to verify this hypothesis.

The present study observed strong MDA5 expression in the epidermis of both dermatomyositis and control skin. This finding indicates that epidermal MDA5 expression itself is not disease-specific. MDA5 belongs to the RIG-I-like receptor family, which interacts with viral RNA and induces cytokine production [25,26]. MDA5 is considered important in the immune response in the skin. A previous study performed immunohistochemical analysis using an anti-human MDA5 polyclonal antibody and reported enhanced expression of MDA5 protein in the epidermis in DM, lichen planus, and chronic discoid lupus erythematosus but not in normal skin [27]. Another study showed that MDA5 expression was significantly increased in the epidermis in psoriasis compared with normal skin [28]. However, these previous studies did not discuss the localisation of MDA5 protein in the epidermis. Our findings suggest that MDA5 acts as an innate immune sensor rather than a disease-specific pathogenic antigen. Its detectability in dermal inflammatory cells in DM reflects cellular localization associated with inflammation rather than increased expression. Herein, we observed that MDA5 protein was strongly expressed in the stratum spinosum and the basal layer of the epidermis in both DM and control skin. In addition to the epidermis, MDA5 immunoreactivity was observed in structures morphologically consistent with dermal capillaries and infiltrating inflammatory cells. Interestingly, MDA5 was barely expressed in the dermis of control skin. To our knowledge, this is the first study to investigate the localisation of MDA5 protein in DM and control skin.

This study has some limitations. First, this study was a small and single-centre cross-sectional study; as such, we were only able to examine skin tissue from a small number of control patients and patients with DM. Although we conducted exploratory and expanded cohort studies using different biopsy cohorts, these limitations remain significant. Second, only immunohistochemical staining was used to examine the deposition of complement proteins and immunoglobulins. Third, the control specimens were obtained from histologically tumor-free skin from DFSP resections rather than from healthy volunteers. Although DFSP was selected because it is routinely excised with a wide 4-cm surgical margin at our institution, these tissues may not be fully equivalent to healthy skin. Local tumor-related effects, anatomical site variation, differences in sun exposure, and other pre-analytic factors may have influenced baseline immunoglobulin and complement staining. In addition, inflammatory skin diseases with interface dermatitis, such as cutaneous lupus erythematosus or lichen planus, were not included as comparator groups. Fourth, direct immunofluorescence, which is the standard method for evaluating immunoglobulin and complement deposition in dermatopathology, was not performed because frozen tissue was not available. In addition, species-matched negative controls were not separately performed for the rabbit primary antibodies. Therefore, nonspecific trapping of serum proteins and vascular leakage cannot be completely excluded. Fifth, detailed information regarding disease duration was not uniformly available because of the retrospective study design and was therefore not included in the present analysis. In addition, information regarding prior use of topical corticosteroids was not consistently available. These factors may have influenced the histopathological findings. In addition, the dermatomyositis cohort was enriched for patients with anti-MDA5 and anti-TIF1-γ antibodies. Therefore, the present findings may not be fully generalizable to patients with other myositis-specific autoantibody subtypes. Formal interobserver agreement was not assessed, and the semi-quantitative scoring system remains inherently subjective despite blinded evaluation and consensus review. Future studies utilising additional analytical approaches will be needed to confirm our hypotheses.

## Conclusion

Our findings revealed strong deposition of complement C3c, IgM, and IgA in interface dermatitis in DM, findings that are consistent with immune complex-mediated injury. Additionally, we demonstrated that the MDA5 protein was expressed in the stratum spinosum and basal layer of the epidermis in both control and DM skin.

## Supporting information

S1 Table Separate immunohistochemical results in the exploratory and expanded cohort studies. (PDF)

S1 File Minimal dataset containing individual clinicopathological and immunohistochemical data used for statistical analyses. (PDF)

## References

[1] Mariampillai K, Granger B, Amelin D, Guiguet M, Hachulla E, Maurier F, et al. Development of a new classification system for idiopathic inflammatory myopathies based on clinical manifestations and myositis-specific autoantibodies. JAMA Neurol. 2018;75(12):1528–37. doi: 10.1001/jamaneurol.2018.2598 30208379 PMC6583199

[2] Sontheimer RD. Cutaneous features of classic dermatomyositis and amyopathic dermatomyositis. Curr Opin Rheumatol. 1999;11(6):475–82. doi: 10.1097/00002281-199911000-00005 10551671

[3] Dalakas MC, Hohlfeld R. Polymyositis and dermatomyositis. Lancet. 2003;362(9388):971–82. doi: 10.1016/S0140-6736(03)14368-1 14511932

[4] Marie I, Hatron PY, Dominique S, Cherin P, Mouthon L, Menard J-F. Short-term and long-term outcomes of interstitial lung disease in polymyositis and dermatomyositis: a series of 107 patients. Arthritis Rheum. 2011;63(11):3439–47. doi: 10.1002/art.30513 21702020

[5] Kondoh Y, Taniguchi H, Kataoka K, Kato K, Suzuki R, Ogura T, et al. Prognostic factors in rapidly progressive interstitial pneumonia. Respirology. 2010;15(2):257–64. doi: 10.1111/j.1440-1843.2009.01687.x 20070584

[6] Sato S, Masui K, Nishina N, Kawaguchi Y, Kawakami A, Tamura M, et al. Initial predictors of poor survival in myositis-associated interstitial lung disease: a multicentre cohort of 497 patients. Rheumatology (Oxford). 2018;57(7):1212–21. doi: 10.1093/rheumatology/key060 29596687

[7] Okamoto M, Yoshida A, Zaizen Y, Ishida M, Shimizu T, Sakamoto N, et al. Gene variants of interferon induced with helicase C domain 1 in Japanese patients with Dermatomyositis-associated rapidly progressive interstitial lung disease: a genetic association study using whole-exome and Sanger sequencing. Respir Res. 2025;26(1):304. doi: 10.1186/s12931-025-03379-3 41174729 PMC12577163

[8] Jerusalem F, Rakusa M, Engel AG, MacDonald RD. Morphometric analysis of skeletal muscle capillary ultrastructure in inflammatory myopathies. J Neurol Sci. 1974;23(3):391–402. doi: 10.1016/0022-510x(74)90157-9 4427123

[9] Carpenter S, Karpati G, Rothman S, Watters G. The childhood type of dermatomyositis. Neurology. 1976;26(10):952–62. doi: 10.1212/wnl.26.10.952 183170

[10] Kissel JT, Mendell JR, Rammohan KW. Microvascular deposition of complement membrane attack complex in dermatomyositis. N Engl J Med. 1986;314(6):329–34. doi: 10.1056/NEJM198602063140601 3945256

[11] Zaizen Y, Okamoto M, Azuma K, Fukuoka J, Hozumi H, Sakamoto N, et al. Enhanced immune complex formation in the lungs of patients with dermatomyositis. Respir Res. 2023;24(1):86. doi: 10.1186/s12931-023-02362-0 36934274 PMC10024827

[12] Bohan A, Peter JB. Polymyositis and dermatomyositis (first of two parts). N Engl J Med. 1975;292(7):344–7. doi: 10.1056/NEJM197502132920706 1090839

[13] Bohan A, Peter JB. Polymyositis and dermatomyositis (second of two parts). N Engl J Med. 1975;292(8):403–7. doi: 10.1056/NEJM197502202920807 1089199

[14] Sontheimer RD. Would a new name hasten the acceptance of amyopathic dermatomyositis (dermatomyositis siné myositis) as a distinctive subset within the idiopathic inflammatory dermatomyopathies spectrum of clinical illness?. J Am Acad Dermatol. 2002;46(4):626–36. doi: 10.1067/mjd.2002.120621 11907524

[15] Okamoto M, Zaizen Y, Kaieda S, Nouno T, Koga T, Matama G, et al. Soluble form of the MDA5 protein in human sera. Heliyon. 2024;10(11):e31727. doi: 10.1016/j.heliyon.2024.e31727 38845920 PMC11153190

[16] Takenaka S-I, Kaieda S, Kawayama T, Matsuoka M, Kaku Y, Kinoshita T, et al. IL-38: A new factor in rheumatoid arthritis. Biochem Biophys Rep. 2015;4:386–91. doi: 10.1016/j.bbrep.2015.10.015 29124228 PMC5669445

[17] Kitasato Y, Hoshino T, Okamoto M, Kato S, Koda Y, Nagata N, et al. Enhanced expression of interleukin-18 and its receptor in idiopathic pulmonary fibrosis. Am J Respir Cell Mol Biol. 2004;31(6):619–25. doi: 10.1165/rcmb.2003-0306OC 15308504

[18] Nouno T, Okamoto M, Ohnishi K, Kaieda S, Tominaga M, Zaizen Y. Elevation of pulmonary CD163. J Thorac Dis. 2019;11(9):4005–17. doi: 10.21037/jtd.2019.09.03 31656675 PMC6790423

[19] Dalakas MC. Immunopathogenesis of inflammatory myopathies. Ann Neurol. 1995;37 Suppl 1:S74-86. doi: 10.1002/ana.410370709 8968219

[20] Vattemi G, Mirabella M, Guglielmi V, Lucchini M, Tomelleri G, Ghirardello A, et al. Muscle biopsy features of idiopathic inflammatory myopathies and differential diagnosis. Auto Immun Highlights. 2014;5(3):77–85. doi: 10.1007/s13317-014-0062-2 26000159 PMC4386579

[21] Jiang D, Ge J, Liao Q, Ma J, Liu Y, Huang J, et al. IgG and IgA with potential microbial-binding activity are expressed by normal human skin epidermal cells. Int J Mol Sci. 2015;16(2):2574–90. doi: 10.3390/ijms16022574 25625513 PMC4346852

[22] Blenkinsopp WK, Clayton RJ, Haffenden GP. Immunoglobulin and complement in normal skin. J Clin Pathol. 1978;31(12):1143–6. doi: 10.1136/jcp.31.12.1143 372244 PMC1145520

[23] Funabiki M, Kato H, Miyachi Y, Toki H, Motegi H, Inoue M, et al. Autoimmune disorders associated with gain of function of the intracellular sensor MDA5. Immunity. 2014;40(2):199–212. doi: 10.1016/j.immuni.2013.12.014 24530055

[24] Lu X, Peng Q, Wang G. Anti-MDA5 antibody-positive dermatomyositis: pathogenesis and clinical progress. Nat Rev Rheumatol. 2024;20(1):48–62. doi: 10.1038/s41584-023-01054-9 38057474

[25] Yoneyama M, Onomoto K, Jogi M, Akaboshi T, Fujita T. Viral RNA detection by RIG-I-like receptors. Curr Opin Immunol. 2015;32:48–53. doi: 10.1016/j.coi.2014.12.012 25594890

[26] Yoneyama M, Fujita T. Function of RIG-I-like receptors in antiviral innate immunity. J Biol Chem. 2007;282(21):15315–8. doi: 10.1074/jbc.R700007200 17395582

[27] Zahn S, Barchet W, Rehkämper C, Hornung T, Bieber T, Tüting T, et al. Enhanced skin expression of melanoma differentiation-associated gene 5 (MDA5) in dermatomyositis and related autoimmune diseases. J Am Acad Dermatol. 2011;64(5):988–9. doi: 10.1016/j.jaad.2010.08.004 21496705

[28] Hong DK, Choi MR, Hwang YL, Lee JK, Lee Y, Seo YJ. Potential Role of Cytosolic RNA Sensor. Ann Dermatol. 2021;33(4):339–44. doi: 10.5021/ad.2021.33.4.339 34341635 PMC8273324

