# Supplementary material for: Expression of melanoma differentiation–associated gene 5 in the epidermis and cutaneous deposition of complement C3 and immunoglobulins in patients with dermatomyositis
Source: PLoS One. 2026 Jun 8;21(6):e0351248. doi: 10.1371/journal.pone.0351248 (PMC13245797; doi:10.1371/journal.pone.0351248)
Supplement: S1 Table — (PDF) [file pone.0351248.s001.pdf]

Expression intensity 0/1/2/3 Median (IQR)	Exploratory study			Expanded cohort study		
	DM (n=6)	control (n=6)	p value	DM (n=16)	control (n=7)	p value
C3c	0/1/1/4<br>3 (2-3)	4/0/2/0<br>0 (0-2)	0.0121*	0/2/6/8<br>3 (2-3)	1/4/1/1<br>1 (1-2)	0.0135*
IgM	0/1/3/2<br>2 (2-3)	3/2/1/0<br>1 (0-1)	0.0163*	2/5/6/3<br>2 (1-2)	4/2/1/0<br>0 (0-1)	0.0219*
IgG	0/0/2/4<br>3 (2-3)	1/0/3/2<br>2 (2-3)	0.2123	0/8/4/4<br>2 (1-3)	1/2/4/0<br>2 (1-2)	0.5671
IgA	0/2/2/2<br>2 (1-3)	1/2/2/1<br>2 (1-2)	0.4029	0/2/3/11<br>3 (2-3)	2/3/2/0<br>1 (0-2)	0.0010*
MDA5	0/0/0/6<br>3 (3-3)	0/0/1/5<br>3 (3-3)	0.3173	0/2/5/9<br>3 (2-3)	0/4/3/0<br>1 (1-2)	0.0062*

* Statistically significant

Abbreviations: DM, dermatomyositis; Ig, immunoglobulin; MDA5, melanoma differentiation-associated gene 5

Expression intensity score 0, negative staining; score 1, weakly positive; score 2, moderately positive; and score 3, strongly positive.
