# Supplementary material for: Expression of melanoma differentiation–associated gene 5 in the epidermis and cutaneous deposition of complement C3 and immunoglobulins in patients with dermatomyositis
Source: PLoS One. 2026 Jun 8;21(6):e0351248. doi: 10.1371/journal.pone.0351248 (PMC13245797; doi:10.1371/journal.pone.0351248)
Supplement: S1 File — (PDF) [file pone.0351248.s002.pdf]

Case No	Age	Sex	Study design	Diagnosis	Bx site	positive Ab	Dx of IIM	C3	IgM	IgG	IgA	MDA5
1	19	F	Exploratory	Dermatomyositis	trunk	Tif-1γ	DM	3	3	3	3	3
2	50	F	Exploratory	Dermatomyositis	upper limb	MDA5	DM	3	2	3	2	3
3	62	M	Exploratory	Dermatomyositis	upper limb	MDA5	CADM	3	1	2	1	3
4	68	M	Exploratory	Dermatomyositis	trunk	Tif-1γ	DM	2	2	3	2	3
5	55	M	Exploratory	Dermatomyositis	lower limb	Tif-1γ	DM	1	2	2	1	3
6	70	F	Exploratory	Dermatomyositis	head	Tif-1γ	DM	3	3	3	3	3
7	47	F	Exploratory	Control	trunk			0	2	2	1	3
8	24	F	Exploratory	Control	trunk			0	0	0	0	3
9	26	F	Exploratory	Control	lower limb			0	0	2	2	3
10	42	F	Exploratory	Control	trunk			2	1	3	3	3
11	46	M	Exploratory	Control	trunk			2	1	3	2	3
12	39	M	Exploratory	Control	trunk			0	0	2	1	2
13	44	M	Expand coh	Dermatomyositis	upper limb	MDA5	CADM	1	0	1	1	1
14	40	F	Expand coh	Dermatomyositis	upper limb	MDA5	DM	2	0	2	2	1
15	52	F	Expand coh	Dermatomyositis	trunk	MDA5	CADM	2	2	3	3	3
16	54	F	Expand coh	Dermatomyositis	trunk	Tif-1γ	DM	3	1	3	3	3
17	71	F	Expand coh	Dermatomyositis	trunk	Tif-1γ	DM	3	1	3	3	2
18	62	M	Expand coh	Dermatomyositis	upper limb	Tif-1γ	DM	1	1	1	1	2
19	88	F	Expand coh	Dermatomyositis	upper limb	Tif-1γ	DM	2	3	2	2	3
20	70	F	Expand coh	Dermatomyositis	head	Tif-1γ	DM	3	2	1	3	2
21	15	F	Expand coh	Dermatomyositis	lower limb	MDA5	DM	2	2	3	3	3
22	42	F	Expand coh	Dermatomyositis	upper limb	Tif-1γ	DM	2	3	1	3	3
23	81	F	Expand coh	Dermatomyositis	upper limb	Tif-1γ	DM	3	2	2	3	3
24	79	M	Expand coh	Dermatomyositis	upper limb	Tif-1γ	DM	2	1	1	3	2
25	65	M	Expand coh	Dermatomyositis	trunk	Tif-1γ	DM	3	1	1	2	3
26	55	M	Expand coh	Dermatomyositis	upper limb	Tif-1γ	DM	3	2	1	3	2
27	79	F	Expand coh	Dermatomyositis	upper limb	Tif-1γ	DM	3	2	2	3	3
28	77	M	Expand coh	Dermatomyositis	upper limb	MDA5	CADM	3	3	1	3	3
29	34	F	Expand coh	Control	trunk			1	0	2	1	1
30	46	M	Expand coh	Control	trunk			0	0	2	2	1
31	35	M	Expand coh	Control	trunk			3	0	2	1	2
32	50	F	Expand coh	Control	trunk			1	1	0	1	1
33	12	F	Expand coh	Control	trunk			2	1	1	0	2
34	55	F	Expand coh	Control	trunk			1	2	2	2	1
35	36	M	Expand coh	Control	trunk			1	0	1	0	2
