# Supplementary material for: Expression of melanoma differentiation–associated gene 5 in the epidermis and cutaneous deposition of complement C3 and immunoglobulins in patients with dermatomyositis
Source: PLoS One. 2026 Jun 8;21(6):e0351248. doi: 10.1371/journal.pone.0351248 (PMC13245797; doi:10.1371/journal.pone.0351248)
Supplement: Attachment — Submitted filename: Reply for previous submission.pdf [file pone.0351248.s003.pdf]

Reviewer #1:

Firstly, the study is based on a small sample size from a limited number of patients and controls, which makes it challenging to generalize the findings. Larger-scale studies are necessary to validate these results.

Reply: We thank the reviewer for this important comment. We considered this issue, which was raised during the previous submission, to be the major limitation of our earlier study. To address this concern, we conducted an expanded cohort study as a separate investigation, increasing the sample size. Based on these results, we have prepared the present new manuscript.

Additionally, being a single-center study, there is a potential for facility-specific staining biases.

Reply: We thank the reviewer for this comment. Although it is difficult to completely exclude the possibility of staining bias, all immunohistochemical staining procedures were performed using the same antibodies and dilution conditions as described in the Materials and Methods section (Immunohistochemical Staining). These conditions were within the ranges recommended in the antibody datasheets. Based on this standardized approach, we therefore consider that institution-specific staining bias is unlikely to have substantially influenced the results.

Furthermore, the study relied solely on immunohistochemical staining to examine the expression of complement proteins and immunoglobulins, without utilizing other analytical methods (e.g., protein analysis or gene expression analysis using skin samples). Incorporating additional methodologies would enhance the reliability of the results.

Reply: We thank the reviewer for this important comment. In the present study, we strengthened the data from the previous submission by conducting an expanded cohort study that included older archival specimens. With regard to the suggested analyses of the proteins and genes, we were concerned that performing such analyses using only older formalin-fixed, paraffin-embedded specimens might introduce additional uncertainty and potentially compromise the reliability of the results. For this reason, we did not perform these evaluations in the current study. We recognize this as a limitation and have explicitly stated it in the final paragraph of the Discussion.

Moreover, the study does not provide direct evidence for the causal relationship between the deposition of complement C3c and IgM and the occurrence of dermatitis. Further research is needed to clarify these relationships.

Reply: We acknowledge that the present study was not designed to establish a causal relationship between cutaneous inflammation and complement or immunoglobulin deposition. To directly demonstrate such relationships, additional analyses—such as protein-based assays, gene expression analyses, or assessments of inflammatory cytokines—would be required. However, as described in our previous response, we were concerned that conducting these analyses using older FFPE specimens could reduce the reliability of the results. Therefore, these analyses were not performed in the present study.

Reviewer #2:
First, the number of cases is very small and there are no comparisons between different subtypes as well as skin lesions of other connective tissue diseases, which may need further validation with more cases.

Reply: We thank the reviewer for this important comment. We considered this issue, which was raised during the previous submission, to be the major limitation of our earlier study. To address this concern, we conducted an expanded cohort study as a separate investigation, increasing the sample size. Based on these results, we have prepared the present new manuscript.

Secondly, the target cells and tissues in the immunohistochemical study of immunoglobulin expression in the dermis were not clear and further clarification would have been appreciated.

Reply: We thank the reviewer for this comment. In dermatomyositis-associated dermatitis, interface dermatitis is a characteristic histopathological feature, accompanied by increased inflammatory cell infiltration and prominent immunoglobulin deposition in this region. We have described these findings in the first paragraph of the Results section entitled "DM skin exhibited higher C3c and immunoglobulin deposition than normal skin."
