# Supplementary material for: Expression of melanoma differentiation–associated gene 5 in the epidermis and cutaneous deposition of complement C3 and immunoglobulins in patients with dermatomyositis
Source: PLoS One. 2026 Jun 8;21(6):e0351248. doi: 10.1371/journal.pone.0351248 (PMC13245797; doi:10.1371/journal.pone.0351248)
Supplement: Attachment — Submitted filename: MDA5_Skin_R1_TH.docx [file pone.0351248.s004.docx]

Reviewer #1:

This manuscript investigates cutaneous deposition of C3c and immunoglobulins and the localization of MDA5 in dermatomyositis (DM) skin using immunohistochemistry. The topic is clinically relevant, and the findings of increased C3c, IgM, and IgA deposition in lesional DM skin, together with the observation of epidermal MDA5 staining in both DM and control skin, are potentially informative. Nevertheless, several issues currently limit the strength of the conclusions, and I therefore recommend major revision.

1. Throughout the manuscript, the control specimens are referred to as "normal skin"; however, these samples were obtained from tumor-free margins of patients with dermatofibrosarcoma protuberans (DFSP). While this is understandable from a practical standpoint, such tissue cannot be assumed to be equivalent to skin from healthy volunteers. Local tumor-related effects, anatomical site variation, differences in sun exposure, and other pre-analytic factors may all influence baseline immunoglobulin and complement staining. The authors should adopt the term "control skin" in place of "normal skin" throughout, provide further detail regarding biopsy site and distance from the tumor margin, and address this limitation more explicitly in the Discussion. Where feasible, the inclusion of healthy volunteer skin and/or inflammatory disease controls exhibiting interface dermatitis—such as cutaneous lupus erythematosus or lichen planus—would substantially strengthen the study.

Authors’ response:

We thank the reviewer for this insightful comment. We agree that the term “normal skin” may be misleading because the control specimens were obtained from histologically tumor-free skin adjacent to DFSP rather than from healthy volunteers. Accordingly, we have replaced the term “normal skin” with “control skin” throughout the manuscript.

We have revised the Methods section to further clarify the rationale for selecting DFSP specimens as controls. At our institution, DFSP is routinely excised with a 4-cm surgical margin, which is the widest margin used for cutaneous tumors. We therefore selected DFSP because it allowed us to obtain histologically tumor-free skin at a substantial distance from the tumor with minimal influence from tumor-associated inflammatory changes.

We agree that these control specimens cannot be considered equivalent to skin from healthy volunteers and that local tumor-related effects, anatomical site variation, sun exposure, and other pre-analytic factors may have influenced baseline immunoglobulin and complement staining. We have added this point to the Discussion as a limitation.

We also agree that inclusion of healthy volunteer skin and inflammatory disease controls with interface dermatitis, such as cutaneous lupus erythematosus or lichen planus, would further strengthen the study. However, these control groups were not included in the original study design and were not covered by the approved study protocol. Therefore, additional analyses using these specimens were beyond the scope of the present revision and should be addressed in future studies.

Based on the reviewer's suggestions, we modified the manuscript as follows:

I corrected most instances where “normal skin” was used (with the exception of phrases such as “normal skin sample as control”) to “control skin.”

(Page 5, line 4) At our institution, DFSP is routinely excised with a 4-cm surgical margin.

(Page 11, line 10) Third, the control specimens were obtained from histologically tumor-free skin from DFSP resections rather than from healthy volunteers. Although DFSP was selected because it is routinely excised with a wide 4-cm surgical margin at our institution, these tissues may not be fully equivalent to healthy skin. Local tumor-related effects, anatomical site variation, differences in sun exposure, and other pre-analytic factors may have influenced baseline immunoglobulin and complement staining. In addition, inflammatory skin diseases with interface dermatitis, such as cutaneous lupus erythematosus or lichen planus, were not included as comparator groups.

2. The present data demonstrate immunohistochemical detection of C3c and immunoglobulins in DM skin, but they do not directly establish immune complex formation or activation of the complement cascade. The conclusion that DM skin is characterized by "immune complex deposition and complement activation" therefore overstates what the evidence supports. The authors should either provide additional supporting data—such as direct immunofluorescence or staining for C4d and/or C5b-9—or revise the language to indicate that the findings are consistent with, rather than confirmatory of, immune complex-mediated injury.

Authors’ response:

We thank the reviewer for this important comment. We agree that immunohistochemical detection of C3c and immunoglobulins does not directly demonstrate immune complex formation or definitive activation of the complement cascade. We did not perform direct immunofluorescence or additional staining for C4d or C5b-9 in the present study.

Accordingly, we have revised the manuscript to avoid overstatement. Specifically, we replaced expressions such as “complement activation” with “complement deposition” and modified the text to indicate that our findings are “consistent with” rather than confirmatory of immune complex-mediated injury.

Based on the reviewer's suggestions, we modified the manuscript as follows:

(Page 2, line 19) DM skin is characterized by the deposition of immunoglobulins and complement C3c at sites of interface dermatitis, findings that are consistent with immune complex-mediated injury.

(Page 10, line 13) These findings are consistent with a possible role of immune complex-mediated injury in the development of dermatitis in DM. Taken together, these findings suggest that skin lesions in dermatomyositis are best characterized as interface dermatitis accompanied by immunoglobulin and complement deposition, findings that are consistent with immune complex-mediated injury rather than nonspecific immunoglobulin accumulation.

(Page 11, line 11) Collectively, these findings suggest that immunoglobulin and complement deposition, together with inflammatory cell infiltration, may contribute to cutaneous inflammation in patients with DM.

(Page 13, line 1) Our findings revealed strong deposition of complement C3c, IgM, and IgA in interface dermatitis in DM, findings that are consistent with immune complex-mediated injury.

3. In dermatopathology, direct immunofluorescence performed on frozen tissue sections is the established method for evaluating immunoglobulin and complement deposition at the dermo-epidermal junction. The authors should explain their rationale for selecting formalin-fixed paraffin-embedded immunohistochemistry, describe how nonspecific serum protein trapping or vascular leakage was excluded, and specify the negative controls used for the rabbit primary antibodies. This concern is particularly relevant given that IgG staining was moderate to strong in both groups, which raises the possibility of background or nonspecific deposition.

Authors’ response:

We thank the reviewer for this important and insightful comment. We agree that direct immunofluorescence using frozen tissue sections is the standard method for evaluating immunoglobulin and complement deposition in dermatopathology. In the present study, however, we used formalin-fixed paraffin-embedded archival specimens because frozen tissue was not available for this retrospective analysis, and all specimens had been collected as part of routine clinical practice.

We have revised the Methods section to clarify the rationale for using immunohistochemistry with formalin-fixed paraffin-embedded tissues. We have also clarified that mouse IgG1 was used as a negative control for our in-house anti-human MDA5 monoclonal antibody (clone H27). We did not perform separate species-matched negative controls for the rabbit primary antibodies against C3c and immunoglobulins, and we acknowledge that this represents a methodological limitation.

In addition, we have expanded the Discussion to note that, because the present study relied on immunohistochemistry rather than direct immunofluorescence, and because species-matched negative controls were not performed for the rabbit primary antibodies, nonspecific trapping of serum proteins and vascular leakage cannot be completely excluded. We also added a brief discussion of the finding that IgG staining was observed in both dermatomyositis and control skin, consistent with previous reports describing baseline immunoglobulin deposition in normal human skin.

Accordingly, we have revised the manuscript as follows to clarify these methodological considerations and to more explicitly acknowledge the limitations of the immunohistochemical approach used in this study.

(Page 7, line 5) Direct immunofluorescence using frozen tissue sections is the standard method for evaluating immunoglobulin and complement deposition in dermatopathology. In the present study, we used formalin-fixed paraffin-embedded archival specimens because frozen tissue was not available for this retrospective analysis and all specimens had been collected as part of routine clinical practice.

(Page 7, line 20) mouse IgG1 (BioLegend Japan, Tokyo, Japan) was used as a negative control for the in-house anti-human MDA5 monoclonal antibody (clone H27). Separate species-matched negative controls were not performed for the rabbit primary antibodies against C3c and immunoglobulins.

(Page 13, line 3) Fourth, direct immunofluorescence, which is the standard method for evaluating immunoglobulin and complement deposition in dermatopathology, was not performed because frozen tissue was not available. In addition, species-matched negative controls were not separately performed for the rabbit primary antibodies. Therefore, nonspecific trapping of serum proteins and vascular leakage cannot be completely excluded.

4. Although a 0–3 ordinal scoring system was employed, the results are reported as mean ± SD, which is not appropriate for ordinal data; median and interquartile range should be reported instead. In addition, the manuscript should specify how many pathologists evaluated the slides, whether scoring was performed independently prior to reaching consensus, and whether interobserver agreement was formally assessed. Reporting the full distribution of scores for each marker, along with exact p-values, would improve transparency. The authors should also clarify whether any correction for multiple comparisons was considered.

Authors’ response:

We thank the reviewer for this important and constructive comment. We agree that, because the immunohistochemical scores were based on an ordinal 0–3 scale, reporting the results as mean ± standard deviation was not the most appropriate approach. Accordingly, we have revised Table 2 to present the data as median (interquartile range) while retaining the full distribution of scores (0/1/2/3) for each marker.

We have also expanded the Methods section to provide additional details regarding the pathological assessment procedure. Specifically, we now state the number of pathologists involved in the evaluation, clarify that all slides were reviewed while blinded to the clinical information, and describe that the final scores were determined by consensus after independent assessment.

In addition, we have revised Table 2 to report numerical p-values rather than threshold expressions whenever possible. Because five immunohistochemical markers were compared between the dermatomyositis and control groups, we additionally performed Bonferroni correction as a sensitivity analysis (adjusted significance threshold, p < 0.01). The significant differences observed for C3c, IgM, and IgA remained statistically significant after correction, confirming the robustness of these findings.

Formal interobserver agreement was not assessed in the present study. We agree that this is an important methodological consideration and have acknowledged in the Discussion that the semi-quantitative scoring system remains inherently subjective despite blinded evaluation and consensus review.

These revisions have been incorporated into the Methods, Results, Table 2, and Discussion sections of the manuscript as follows:

(Page 8, line 9) Three evaluators (YZ, MT, and JA) independently reviewed all slides while blinded to clinical information, and the final scores were determined by consensus.

(Page 8, line 14) Because five immunohistochemical markers were compared between groups, Bonferroni correction was additionally performed as a sensitivity analysis. A p value of <0.01 was considered statistically significant after correction.

(Page 13, line 13) Formal interobserver agreement was not assessed, and the semi-quantitative scoring system remains inherently subjective despite blinded evaluation and consensus review.

5. The manuscript indicates that the expanded cohort was assembled following the initial observations and that the two cohorts were subsequently combined. This approach requires clarification. If the expanded cohort was recruited after reviewing the exploratory results, pooling the data may introduce bias, and it should be stated explicitly whether this pooled analysis was prespecified. At a minimum, results from the two cohorts should be presented separately, even if a combined analysis is retained, in order to demonstrate consistency across cohorts.

Authors’ response:

We thank the reviewer for this insightful comment. We agree that the relationship between the exploratory study and the expanded cohort study required further clarification.

The expanded cohort study was conducted to confirm the observations obtained in the exploratory study using the same inclusion criteria, immunohistochemical procedures, semi-quantitative scoring system, and statistical methods. As originally planned, the final analysis was performed using the pooled dataset after confirming that the overall staining patterns and group differences were generally consistent between the two cohorts.

To improve transparency, we have revised the Methods section to clarify this study design. In addition, we have provided the results of the exploratory study and the expanded cohort study separately in Supporting Information (S1 Table), while retaining the pooled analysis as the primary analysis.

In the expanded cohort, MDA5 staining scores were significantly lower in control skin than in dermatomyositis skin. However, several control specimens in this cohort were older archival samples, and reduced staining intensity related to long-term storage may have contributed to this finding. We have added this point to the manuscript as follows:

(Page 6, line 12) The expanded cohort was assembled to confirm the observations obtained in the exploratory study using the same immunohistochemical and statistical methods. As originally planned, the final analysis was performed using the pooled dataset after confirming that the findings were directionally consistent between the two cohorts.

(Page 10, line 3) The results of the exploratory study and the expanded cohort study analyzed separately are provided in S1 Table. Overall staining patterns and group differences were generally consistent between the two cohorts. In the expanded cohort, MDA5 staining scores were significantly lower in control skin than in dermatomyositis skin; however, several control specimens were older archival samples, and reduced staining intensity related to long-term storage may have contributed to this finding.

6. The authors note that MDA5 staining appeared relatively weak in several older specimens, raising concern about pre-analytic variability more broadly. The manuscript should report, where not already provided, the distribution of specimen age, fixation conditions, anatomical biopsy site, disease duration, and treatment status at the time of biopsy. In particular, prior use of systemic corticosteroids or immunosuppressive therapy could influence both inflammatory infiltrates and protein deposition and should therefore be described and discussed.

Authors’ response:

We thank the reviewer for this important comment. We agree that pre-analytic factors and treatment status may influence immunohistochemical staining intensity and should be described more clearly.

We note that several of the requested variables were already included in the original manuscript. Specifically, the biopsy sites are presented in Table 1, the specimen collection periods are described in the Methods section, and all tissues were processed as formalin-fixed paraffin-embedded specimens using the same immunohistochemical protocol.

Importantly, none of the patients with dermatomyositis had received systemic corticosteroids or immunosuppressive agents at the time of skin biopsy. We have added this information to the Methods section. Information regarding prior use of topical corticosteroids was not consistently available because of the retrospective study design and therefore could not be analyzed systematically.

Detailed information regarding disease duration was not uniformly available and was not included in the present analysis. We agree that this is a limitation and have acknowledged this point in the Discussion.

In addition, we have expanded the Discussion to note that several control specimens were older archival samples and that long-term storage of formalin-fixed paraffin-embedded tissues may have influenced staining intensity, particularly for MDA5.

Accordingly, we have revised the Methods and Discussion sections to more explicitly describe these pre-analytic and clinical factors and their potential impact on the results.

(Page 5, line 24) None of the patients with dermatomyositis had received systemic corticosteroids or immunosuppressive agents at the time of skin biopsy.

(Page 13, line 25) Fifth, detailed information regarding disease duration was not uniformly available because of the retrospective study design and was therefore not included in the present analysis. In addition, information regarding prior use of topical corticosteroids was not consistently available. These factors may have influenced the histopathological findings.

7. The finding of prominent epidermal MDA5 expression in both DM and control skin is of interest; however, the conclusions regarding MDA5-positive dermal capillaries and inflammatory cells require stronger support. The Methods indicate that scoring focused on the superficial dermis near the dermo-epidermal junction, yet statements about epidermal expression are also made. The authors should clarify whether epidermal and dermal compartments were evaluated separately. If dermal localization constitutes the primary novel finding, compartment-specific quantification and/or co-staining with endothelial and inflammatory cell markers would substantially strengthen this conclusion. Additional validation of the in-house H27 antibody in skin tissue would likewise be informative.

Authors’ response:

We thank the reviewer for this helpful comment. We agree that the identification of dermal capillaries and inflammatory cells was based on morphological assessment using single-marker immunohistochemistry, and that the precise cellular origin of MDA5 immunoreactivity could not be definitively established without double staining or lineage-specific markers.

Accordingly, we have revised the Results and Discussion to state that MDA5 immunoreactivity was observed in structures morphologically consistent with dermal capillaries and infiltrating inflammatory cells, rather than making definitive statements regarding specific cell types. We have also added this point to the Discussion.

(Page 9, line 19) In DM skin, MDA5 immunoreactivity was also observed in structures morphologically consistent with dermal capillaries and infiltrating inflammatory cells

(Page 13, line 7) In addition to the epidermis, MDA5 immunoreactivity was observed in structures morphologically consistent with dermal capillaries and infiltrating inflammatory cells.

8. The DM cohort appears to include anti-MDA5-positive and anti-TIF1-γ-positive cases but no anti-ARS-positive or anti-Mi-2-positive cases. This restricts the generalizability of the findings to DM as a whole. The authors should address this limitation explicitly and avoid broad statements that imply uniformity across all DM subsets. Where feasible, subgroup analyses stratified by autoantibody status would add value, particularly given that MDA5-associated disease mechanisms may differ from those in other serological subsets.

Authors’ response:

We thank the reviewer for this important comment. We agree that the dermatomyositis cohort in this study was enriched for patients with anti-MDA5 and anti-TIF1-γ antibodies, and therefore may not fully represent the entire clinical and serological spectrum of dermatomyositis.

Accordingly, we have added this point to the Discussion as a limitation and now state that the generalizability of our findings to patients with other myositis-specific autoantibody subtypes remains to be determined in future studies.

(Page 14, line 8) In addition, the dermatomyositis cohort was enriched for patients with anti-MDA5 and anti-TIF1-γ antibodies. Therefore, the present findings may not be fully generalizable to patients with other myositis-specific autoantibody subtypes.

9. Several statements in the Discussion extend beyond what the presented data can support. In particular, the proposed links among immune deposition, inflammatory infiltrates, interferon production, and "allergic reactions" are speculative and were not directly examined in this study. The Discussion would benefit from a clearer delineation between findings substantiated by the present data and hypotheses that require future investigation.

Authors’ response:

We thank the reviewer for this helpful comment. We agree that portions of the Discussion were overly speculative and extended beyond the findings directly supported by the present study.

Accordingly, we have revised the Discussion to remove statements regarding allergic reactions, interferon production, and other mechanistic hypotheses that were not directly examined. The revised text focuses on the descriptive findings of immunoglobulin and complement deposition and MDA5 expression and their potential relevance to cutaneous inflammation in dermatomyositis.

Please also refer to our response to Comment 2.

10. The manuscript states that patients met the Bohan and Peter criteria. Because contemporary DM cohorts—particularly those enriched for anti-MDA5-positive cases—frequently include clinically amyopathic presentations, the authors should clarify whether such patients were included or excluded and whether a more recently proposed classification framework was considered.

Authors’ response:

We thank the reviewer for this important comment. We agree that clinically amyopathic dermatomyositis (CADM) may not be fully encompassed by the original Bohan and Peter criteria. In the present study, four of the 22 patients had CADM.

To clarify this point, we have revised the Methods section to state that patients were diagnosed by experienced clinicians based on characteristic cutaneous manifestations, serological findings, and established diagnostic criteria, and that clinically amyopathic cases were included when the diagnosis of dermatomyositis was considered appropriate. We have also added the number of CADM cases to Table 1.

Reviewer #2:

1. Control group restricted to DFSP margin skin; no inflammatory disease comparators. Clarify rationale for DFSP selection in Methods and discuss how this choice may affect specificity and interpretation.

Authors’ response:

We thank the reviewer for this helpful comment. This issue was also raised by Reviewer #1 (Comment 1). We have revised the Methods section to clarify the rationale for selecting DFSP specimens as controls and expanded the Discussion to discuss the limitations associated with this control group. Please refer to our detailed response to Reviewer #1, Comment 1.

2. Reliance on semi quantitative IHC scoring (subjective). Describe scoring procedure in greater detail (blinding, consensus process), report inter rater agreement if available and note scoring limitations in the Discussion.

Authors’ response:

We thank the reviewer for this helpful comment. This issue was also raised by Reviewer #1 (Comment 4). As described in our response to that comment, we have revised the manuscript to provide additional details regarding the semi-quantitative scoring procedure, including blinded independent evaluation by three pathologists followed by consensus review. We have also revised Table 2 to present the results as median (interquartile range), report numerical p-values, and added Bonferroni correction as a sensitivity analysis. In addition, we have acknowledged in the Discussion that formal interobserver agreement was not assessed and that the semi-quantitative scoring system remains inherently subjective despite blinded evaluation and consensus review.

Please refer to our detailed response to Reviewer #1, Comment 4.

3. Limited orthogonal validation and antibody validation details reported. Add a brief methods subsection describing validation of the in house anti MDA5 mAb (e.g., prior publications, specificity controls used) and state any known limitations.

Authors’ response:

We thank the reviewer for this important and helpful comment. We agree that additional information regarding the validation of our in-house anti-human MDA5 monoclonal antibody (clone H27) would strengthen the manuscript.

This issue was addressed by expanding the Methods section describing the establishment of the H27 anti-human monoclonal antibody. We now clarify that this monoclonal antibody was generated using recombinant full-length human MDA5 protein as the immunogen and that its development and application have been reported previously in our studies of dermatomyositis-associated interstitial lung disease and soluble MDA5 protein. In situ analysis revealed that H27 can detect human MDA5 protein in regions where human MDA5 mRNA was strongly expressed. In addition, H27 can detect human MDA5 but not mouse MDA5 protein [11, 15]. We have also clarified that mouse IgG1 was used as a negative control in the present study.

We acknowledge that additional orthogonal validation in skin tissue was not performed in the present study. This represents a methodological limitation and should be considered when interpreting the results.

Accordingly, we have revised the manuscript as follows:

(Page 7, line 7) Expanded description of the establishment of the in-house anti-human MDA5 monoclonal antibody (clone H27), including the use of recombinant full-length human MDA5 protein as the immunogen and references to our previous validation studies [11, 15].

(Page 8, line 1) mouse IgG1 was used as a negative control for the in-house anti-human MDA5 monoclonal antibody (clone H27).

4. Cell type localization descriptions are qualitative. Improve wording to more precisely describe observed cellular localization (e.g., “MDA5 immunoreactivity was observed predominantly in spinous and basal keratinocytes and in dermal inflammatory cells and capillaries”) and acknowledge limits of IHC resolution.

Authors’ response:

We thank the reviewer for this helpful comment. This issue was also raised by Reviewer #1 (Comment 7). As described in our response to that comment, we have revised the Results and Discussion to more precisely describe the observed localization of MDA5 immunoreactivity. Specifically, we now state that MDA5 was strongly expressed in the stratum spinosum and basal layer of the epidermis in both dermatomyositis and control skin, and that additional MDA5 immunoreactivity was observed in structures morphologically consistent with dermal capillaries and infiltrating inflammatory cells in dermatomyositis skin.

We also acknowledge that the identification of these dermal structures was based on morphological assessment using single-marker immunohistochemistry, and that the precise cellular origin of MDA5 immunoreactivity could not be definitively established without double staining or lineage-specific markers.

Please refer to our detailed response to Reviewer #1, Comment 7.

5. Please add the following study in the (comparative context): Similar patterns of immunoglobulin and complement deposition have been reported in pemphigus, where tissue Ig and complement levels correlate with disease activity (Elhag et al., 2024), consistent with our observation of C3c, IgM and IgA deposition in DM interface dermatitis.

-Elhag, Omer Osman, et al. "Association of immunoglobulins and complement levels with pemphigus in patients at the Khartoum Dermatology and Venereal Disease Hospital, Sudan." Italian Journal of Medicine 18.4 (2024). 10.4081/itjm.2024.1831

Authors’ response:

We thank the reviewer for this helpful suggestion. We agree that reports describing immunoglobulin and complement deposition in other autoimmune skin diseases provide useful context for interpreting our findings.

We carefully reviewed the article suggested by the reviewer. However, because pemphigus is a distinct autoimmune blistering disease with a fundamentally different pathogenesis and histopathological pattern from dermatomyositis, we believe that a detailed discussion of this study would have limited relevance to the central findings of the present work. In addition, in light of the editor's request to maintain a concise and focused Discussion, we elected not to incorporate this reference into the revised manuscript.

We hope that the reviewer will understand our decision to keep the Discussion focused on studies that are more directly related to the pathogenesis of dermatomyositis.

6. Limited correlation between histology and clinical variables presented. Present available clinical correlations (antibody status, biopsy site, age, treatment) in Results or a Supplementary table and note any observed trends or lack thereof.

Authors’ response:

We thank the reviewer for this insightful comment. We agree that correlations between histological findings and clinical variables are of interest.

In the present study, relevant clinical variables, including age, biopsy site, and autoantibody status, are summarized in Table 1. We also added information to the Methods section indicating that none of the patients had received systemic corticosteroids or immunosuppressive agents at the time of skin biopsy. In the Results, we state that strong epidermal MDA5 expression was observed in all dermatomyositis cases regardless of anti-MDA5 antibody status.

Because of the limited sample size and the exploratory nature of this study, formal subgroup analyses and correlation testing between clinical variables and immunohistochemical scores were not performed. No clear clinicopathological trends were identified beyond the observation that epidermal MDA5 expression was consistently present irrespective of anti-MDA5 antibody positivity.

We have clarified these points in the revised manuscript and acknowledge that larger studies will be required to further investigate clinicopathological correlations.

7. Potential effects of specimen age/archival storage on staining not fully described. State specimen age/storage ranges in Methods and comment in Limitations how archival status may have influenced staining intensity.

Authors’ response:

We thank the reviewer for this important comment. This issue was also raised by Reviewer #1 (Comment 6). As described in our response to that comment, we have clarified several pre-analytic and clinical factors that may influence immunohistochemical staining intensity.

Specifically, we have added to the Methods section that none of the patients with dermatomyositis had received systemic corticosteroids or immunosuppressive agents at the time of skin biopsy. We also clarified in the Discussion that detailed information regarding disease duration was not uniformly available, that information regarding prior use of topical corticosteroids was not consistently available, and that several control specimens were older archival samples, which may have influenced staining intensity.

Please refer to our detailed response to Reviewer #1, Comment 6.
