# Supplementary material for: Expression of melanoma differentiation–associated gene 5 in the epidermis and cutaneous deposition of complement C3 and immunoglobulins in patients with dermatomyositis
Source: PLoS One. 2026 Jun 8;21(6):e0351248. doi: 10.1371/journal.pone.0351248 (PMC13245797; doi:10.1371/journal.pone.0351248)
Supplement: Peer review history

# Author comment: Author response to Decision Letter 0

**DOI:** 10.1371/journal.pone.0351248.r001

9 Feb 2026

Attachment Submitted filename: Reply for previous submission.pdf

# Peer review: Decision Letter 0

**DOI:** 10.1371/journal.pone.0351248.r002

**Authors:** Seth Domfeh

4 May 2026

PONE-D-26-04603

Expression of Melanoma Differentiation–Associated Gene 5 in the Epidermis and Cutaneous Deposition of Complement C3 and Immunoglobulins in Patients with Dermatomyositis

PLOS One

Dear Dr. Hoshino,

Thank you for submitting your manuscript to PLOS ONE. After careful consideration, we feel that it has merit but does not fully meet PLOS ONE’s publication criteria as it currently stands. Therefore, we invite you to submit a revised version of the manuscript that addresses the points raised during the review process.

Please submit your revised manuscript by Jun 18 2026 11:59PM. If you will need more time than this to complete your revisions, please reply to this message or contact the journal office at plosone@plos.org. When you're ready to submit your revision, log on to https://www.editorialmanager.com/pone/ and select the 'Submissions Needing Revision' folder to locate your manuscript file.

Please include the following items when submitting your revised manuscript:

- A letter that responds to each point raised by the academic editor and reviewer(s). You should upload this letter as a separate file labeled 'Response to Reviewers'.
- A marked-up copy of your manuscript that highlights changes made to the original version. You should upload this as a separate file labeled 'Revised Manuscript with Track Changes'.
- An unmarked version of your revised paper without tracked changes. You should upload this as a separate file labeled 'Manuscript'.

If you would like to make changes to your financial disclosure, please include your updated statement in your cover letter. Guidelines for resubmitting your figure files are available below the reviewer comments at the end of this letter.

If applicable, we recommend that you deposit your laboratory protocols in protocols.io to enhance the reproducibility of your results. Protocols.io assigns your protocol its own identifier (DOI) so that it can be cited independently in the future. For instructions see: https://journals.plos.org/plosone/s/submission-guidelines#loc-laboratory-protocols. Additionally, PLOS ONE offers an option for publishing peer-reviewed Lab Protocol articles, which describe protocols hosted on protocols.io. Read more information on sharing protocols at https://plos.org/protocols?utm_medium=editorial-email&utm_source=authorletters&utm_campaign=protocols.

As the corresponding author, your ORCID iD is verified in the submission system and will appear in the published article. PLOS supports the use of ORCID, and we encourage all coauthors to register for an ORCID iD and use it as well. Please encourage your coauthors to verify their ORCID iD within the submission system before final acceptance, as unverified ORCID iDs will not appear in the published article. *Only* the individual author can complete the verification step; PLOS staff *cannot* verify ORCID iDs on behalf of authors.

We look forward to receiving your revised manuscript.

Kind regards,

Seth Agyei Domfeh, PhD

Academic Editor

PLOS One

Journal Requirements:

When submitting your revision, we need you to address these additional requirements.

1. Please ensure that your manuscript meets PLOS ONE's style requirements, including those for file naming. The PLOS ONE style templates can be found at

https://journals.plos.org/plosone/s/file?id=wjVg/PLOSOne_formatting_sample_main_body.pdf and

https://journals.plos.org/plosone/s/file?id=ba62/PLOSOne_formatting_sample_title_authors_affiliations.pdf

2. Thank you for stating the following in the Acknowledgments Section of your manuscript:

“Funding Declaration: This work was supported by JSPS KAKENHI Grant Numbers JP23K15221, JP25K19452.”

We note that you have provided additional information within the Acknowledgements Section that is not currently declared in your Funding Statement. Please note that funding information should not appear in the Acknowledgments section or other areas of your manuscript. We will only publish funding information present in the Funding Statement section of the online submission form.

Please remove any funding-related text from the manuscript and let us know how you would like to update your Funding Statement. Currently, your Funding Statement reads as follows:

“This work was supported by JSPS KAKENHI Grant Numbers JP23K15221, JP25K19452.”

Please include your amended statements within your cover letter; we will change the online submission form on your behalf.

3. Thank you for stating the following financial disclosure:

“This work was supported by JSPS KAKENHI Grant Numbers JP23K15221, JP25K19452.”

Please state what role the funders took in the study.  If the funders had no role, please state: "The funders had no role in study design, data collection and analysis, decision to publish, or preparation of the manuscript."

If this statement is not correct you must amend it as needed.

Please include this amended Role of Funder statement in your cover letter; we will change the online submission form on your behalf.

4. We note that your Data Availability Statement is currently as follows: “All relevant data are within the manuscript and its Supporting Information files.”

Please confirm at this time whether or not your submission contains all raw data required to replicate the results of your study. Authors must share the “minimal data set” for their submission. PLOS defines the minimal data set to consist of the data required to replicate all study findings reported in the article, as well as related metadata and methods (https://journals.plos.org/plosone/s/data-availability#loc-minimal-data-set-definition).

For example, authors should submit the following data:

- The values behind the means, standard deviations and other measures reported;

- The values used to build graphs;

- The points extracted from images for analysis.

Authors do not need to submit their entire data set if only a portion of the data was used in the reported study.

If your submission does not contain these data, please either upload them as Supporting Information files or deposit them to a stable, public repository and provide us with the relevant URLs, DOIs, or accession numbers. For a list of recommended repositories, please see https://journals.plos.org/plosone/s/recommended-repositories.

If there are ethical or legal restrictions on sharing a de-identified data set, please explain them in detail (e.g., data contain potentially sensitive information, data are owned by a third-party organization, etc.) and who has imposed them (e.g., an ethics committee). Please also provide contact information for a data access committee, ethics committee, or other institutional body to which data requests may be sent. If data are owned by a third party, please indicate how others may request data access.

5. If the reviewer comments include a recommendation to cite specific previously published works, please review and evaluate these publications to determine whether they are relevant and should be cited. There is no requirement to cite these works unless the editor has indicated otherwise.

[Note: HTML markup is below. Please do not edit.]

Reviewers' comments:

Reviewer's Responses to Questions

**Comments to the Author**

1. Is the manuscript technically sound, and do the data support the conclusions?

The manuscript must describe a technically sound piece of scientific research with data that supports the conclusions. Experiments must have been conducted rigorously, with appropriate controls, replication, and sample sizes. The conclusions must be drawn appropriately based on the data presented.

Reviewer #1: Partly

Reviewer #2: Yes

**********

2. Has the statistical analysis been performed appropriately and rigorously?

Reviewer #1: Yes

Reviewer #2: Yes

**********

3. Have the authors made all data underlying the findings in their manuscript fully available?

The PLOS Data policy requires authors to make all data underlying the findings described in their manuscript fully available without restriction, with rare exception (please refer to the Data Availability Statement in the manuscript PDF file). The data should be provided as part of the manuscript or its supporting information, or deposited to a public repository. For example, in addition to summary statistics, the data points behind means, medians and variance measures should be available. If there are restrictions on publicly sharing data—e.g. participant privacy or use of data from a third party—those must be specified.

Reviewer #1: Yes

Reviewer #2: Yes

**********

4. Is the manuscript presented in an intelligible fashion and written in standard English?

PLOS ONE does not copyedit accepted manuscripts, so the language in submitted articles must be clear, correct, and unambiguous. Any typographical or grammatical errors should be corrected at revision, so please note any specific errors here.

Reviewer #1: Yes

Reviewer #2: Yes

**********

5. Review Comments to the Author

Please use the space provided to explain your answers to the questions above. You may also include additional comments for the author, including concerns about dual publication, research ethics, or publication ethics. (Please upload your review as an attachment if it exceeds 20,000 characters)

Reviewer #1: This manuscript investigates cutaneous deposition of C3c and immunoglobulins and the localization of MDA5 in dermatomyositis (DM) skin using immunohistochemistry. The topic is clinically relevant, and the findings of increased C3c, IgM, and IgA deposition in lesional DM skin, together with the observation of epidermal MDA5 staining in both DM and control skin, are potentially informative. Nevertheless, several issues currently limit the strength of the conclusions, and I therefore recommend major revision.

1. Throughout the manuscript, the control specimens are referred to as "normal skin"; however, these samples were obtained from tumor-free margins of patients with dermatofibrosarcoma protuberans (DFSP). While this is understandable from a practical standpoint, such tissue cannot be assumed to be equivalent to skin from healthy volunteers. Local tumor-related effects, anatomical site variation, differences in sun exposure, and other pre-analytic factors may all influence baseline immunoglobulin and complement staining. The authors should adopt the term "control skin" in place of "normal skin" throughout, provide further detail regarding biopsy site and distance from the tumor margin, and address this limitation more explicitly in the Discussion. Where feasible, the inclusion of healthy volunteer skin and/or inflammatory disease controls exhibiting interface dermatitis—such as cutaneous lupus erythematosus or lichen planus—would substantially strengthen the study.

2. The present data demonstrate immunohistochemical detection of C3c and immunoglobulins in DM skin, but they do not directly establish immune complex formation or activation of the complement cascade. The conclusion that DM skin is characterized by "immune complex deposition and complement activation" therefore overstates what the evidence supports. The authors should either provide additional supporting data—such as direct immunofluorescence or staining for C4d and/or C5b-9—or revise the language to indicate that the findings are consistent with, rather than confirmatory of, immune complex-mediated injury.

3. In dermatopathology, direct immunofluorescence performed on frozen tissue sections is the established method for evaluating immunoglobulin and complement deposition at the dermo-epidermal junction. The authors should explain their rationale for selecting formalin-fixed paraffin-embedded immunohistochemistry, describe how nonspecific serum protein trapping or vascular leakage was excluded, and specify the negative controls used for the rabbit primary antibodies. This concern is particularly relevant given that IgG staining was moderate to strong in both groups, which raises the possibility of background or nonspecific deposition.

4. Although a 0–3 ordinal scoring system was employed, the results are reported as mean ± SD, which is not appropriate for ordinal data; median and interquartile range should be reported instead. In addition, the manuscript should specify how many pathologists evaluated the slides, whether scoring was performed independently prior to reaching consensus, and whether interobserver agreement was formally assessed. Reporting the full distribution of scores for each marker, along with exact p-values, would improve transparency. The authors should also clarify whether any correction for multiple comparisons was considered.

5. The manuscript indicates that the expanded cohort was assembled following the initial observations and that the two cohorts were subsequently combined. This approach requires clarification. If the expanded cohort was recruited after reviewing the exploratory results, pooling the data may introduce bias, and it should be stated explicitly whether this pooled analysis was prespecified. At a minimum, results from the two cohorts should be presented separately, even if a combined analysis is retained, in order to demonstrate consistency across cohorts.

6. The authors note that MDA5 staining appeared relatively weak in several older specimens, raising concern about pre-analytic variability more broadly. The manuscript should report, where not already provided, the distribution of specimen age, fixation conditions, anatomical biopsy site, disease duration, and treatment status at the time of biopsy. In particular, prior use of systemic corticosteroids or immunosuppressive therapy could influence both inflammatory infiltrates and protein deposition and should therefore be described and discussed.

7. The finding of prominent epidermal MDA5 expression in both DM and control skin is of interest; however, the conclusions regarding MDA5-positive dermal capillaries and inflammatory cells require stronger support. The Methods indicate that scoring focused on the superficial dermis near the dermo-epidermal junction, yet statements about epidermal expression are also made. The authors should clarify whether epidermal and dermal compartments were evaluated separately. If dermal localization constitutes the primary novel finding, compartment-specific quantification and/or co-staining with endothelial and inflammatory cell markers would substantially strengthen this conclusion. Additional validation of the in-house H27 antibody in skin tissue would likewise be informative.

8. The DM cohort appears to include anti-MDA5-positive and anti-TIF1-γ-positive cases but no anti-ARS-positive or anti-Mi-2-positive cases. This restricts the generalizability of the findings to DM as a whole. The authors should address this limitation explicitly and avoid broad statements that imply uniformity across all DM subsets. Where feasible, subgroup analyses stratified by autoantibody status would add value, particularly given that MDA5-associated disease mechanisms may differ from those in other serological subsets.

9. Several statements in the Discussion extend beyond what the presented data can support. In particular, the proposed links among immune deposition, inflammatory infiltrates, interferon production, and "allergic reactions" are speculative and were not directly examined in this study. The Discussion would benefit from a clearer delineation between findings substantiated by the present data and hypotheses that require future investigation.

10. The manuscript states that patients met the Bohan and Peter criteria. Because contemporary DM cohorts—particularly those enriched for anti-MDA5-positive cases—frequently include clinically amyopathic presentations, the authors should clarify whether such patients were included or excluded and whether a more recently proposed classification framework was considered.

Reviewer #2: 1. Control group restricted to DFSP margin skin; no inflammatory disease comparators. Clarify rationale for DFSP selection in Methods and discuss how this choice may affect specificity and interpretation.

2. Reliance on semi quantitative IHC scoring (subjective). Describe scoring procedure in greater detail (blinding, consensus process), report inter rater agreement if available and note scoring limitations in the Discussion.

3. Limited orthogonal validation and antibody validation details reported. Add a brief methods subsection describing validation of the in house anti MDA5 mAb (e.g., prior publications, specificity controls used) and state any known limitations.

4. Cell type localization descriptions are qualitative. Improve wording to more precisely describe observed cellular localization (e.g., “MDA5 immunoreactivity was observed predominantly in spinous and basal keratinocytes and in dermal inflammatory cells and capillaries”) and acknowledge limits of IHC resolution.

5. Please add the following study in the (comparative context): Similar patterns of immunoglobulin and complement deposition have been reported in pemphigus, where tissue Ig and complement levels correlate with disease activity (Elhag et al., 2024), consistent with our observation of C3c, IgM and IgA deposition in DM interface dermatitis.

-Elhag, Omer Osman, et al. "Association of immunoglobulins and complement levels with pemphigus in patients at the Khartoum Dermatology and Venereal Disease Hospital, Sudan." Italian Journal of Medicine 18.4 (2024). 10.4081/itjm.2024.1831

6. Limited correlation between histology and clinical variables presented. Present available clinical correlations (antibody status, biopsy site, age, treatment) in Results or a Supplementary table and note any observed trends or lack thereof.

7. Potential effects of specimen age/archival storage on staining not fully described. State specimen age/storage ranges in Methods and comment in Limitations how archival status may have influenced staining intensity.

**********

6. PLOS authors have the option to publish the peer review history of their article (what does this mean?). If published, this will include your full peer review and any attached files.

If you choose “no”, your identity will remain anonymous but your review may still be made public.

**Do you want your identity to be public for this peer review?** For information about this choice, including consent withdrawal, please see our Privacy Policy.

Reviewer #1: No

Reviewer #2: No

**********

[NOTE: If reviewer comments were submitted as an attachment file, they will be attached to this email and accessible via the submission site. Please log into your account, locate the manuscript record, and check for the action link "View Attachments". If this link does not appear, there are no attachment files.]

To ensure your figures meet our technical requirements, please review our figure guidelines: https://journals.plos.org/plosone/s/figures

You may also use PLOS’s free figure tool, NAAS, to help you prepare publication quality figures: https://journals.plos.org/plosone/s/figures#loc-tools-for-figure-preparation.

NAAS will assess whether your figures meet our technical requirements by comparing each figure against our figure specifications.

# Author comment: Author response to Decision Letter 1

**DOI:** 10.1371/journal.pone.0351248.r003

10 May 2026

Reviewer #1:

This manuscript investigates cutaneous deposition of C3c and immunoglobulins and the localization of MDA5 in dermatomyositis (DM) skin using immunohistochemistry. The topic is clinically relevant, and the findings of increased C3c, IgM, and IgA deposition in lesional DM skin, together with the observation of epidermal MDA5 staining in both DM and control skin, are potentially informative. Nevertheless, several issues currently limit the strength of the conclusions, and I therefore recommend major revision.

1. Throughout the manuscript, the control specimens are referred to as "normal skin"; however, these samples were obtained from tumor-free margins of patients with dermatofibrosarcoma protuberans (DFSP). While this is understandable from a practical standpoint, such tissue cannot be assumed to be equivalent to skin from healthy volunteers. Local tumor-related effects, anatomical site variation, differences in sun exposure, and other pre-analytic factors may all influence baseline immunoglobulin and complement staining. The authors should adopt the term "control skin" in place of "normal skin" throughout, provide further detail regarding biopsy site and distance from the tumor margin, and address this limitation more explicitly in the Discussion. Where feasible, the inclusion of healthy volunteer skin and/or inflammatory disease controls exhibiting interface dermatitis—such as cutaneous lupus erythematosus or lichen planus—would substantially strengthen the study.

Authors’ response:

We thank the reviewer for this insightful comment. We agree that the term “normal skin” may be misleading because the control specimens were obtained from histologically tumor-free skin adjacent to DFSP rather than from healthy volunteers. Accordingly, we have replaced the term “normal skin” with “control skin” throughout the manuscript.

We have revised the Methods section to further clarify the rationale for selecting DFSP specimens as controls. At our institution, DFSP is routinely excised with a 4-cm surgical margin, which is the widest margin used for cutaneous tumors. We therefore selected DFSP because it allowed us to obtain histologically tumor-free skin at a substantial distance from the tumor with minimal influence from tumor-associated inflammatory changes.

We agree that these control specimens cannot be considered equivalent to skin from healthy volunteers and that local tumor-related effects, anatomical site variation, sun exposure, and other pre-analytic factors may have influenced baseline immunoglobulin and complement staining. We have added this point to the Discussion as a limitation.

We also agree that inclusion of healthy volunteer skin and inflammatory disease controls with interface dermatitis, such as cutaneous lupus erythematosus or lichen planus, would further strengthen the study. However, these control groups were not included in the original study design and were not covered by the approved study protocol. Therefore, additional analyses using these specimens were beyond the scope of the present revision and should be addressed in future studies.

Based on the reviewer's suggestions, we modified the manuscript as follows:

I corrected most instances where “normal skin” was used (with the exception of phrases such as “normal skin sample as control”) to “control skin.”

(Page 5, line 4) At our institution, DFSP is routinely excised with a 4-cm surgical margin.

(Page 11, line 10) Third, the control specimens were obtained from histologically tumor-free skin from DFSP resections rather than from healthy volunteers. Although DFSP was selected because it is routinely excised with a wide 4-cm surgical margin at our institution, these tissues may not be fully equivalent to healthy skin. Local tumor-related effects, anatomical site variation, differences in sun exposure, and other pre-analytic factors may have influenced baseline immunoglobulin and complement staining. In addition, inflammatory skin diseases with interface dermatitis, such as cutaneous lupus erythematosus or lichen planus, were not included as comparator groups.

2. The present data demonstrate immunohistochemical detection of C3c and immunoglobulins in DM skin, but they do not directly establish immune complex formation or activation of the complement cascade. The conclusion that DM skin is characterized by "immune complex deposition and complement activation" therefore overstates what the evidence supports. The authors should either provide additional supporting data—such as direct immunofluorescence or staining for C4d and/or C5b-9—or revise the language to indicate that the findings are consistent with, rather than confirmatory of, immune complex-mediated injury.

Authors’ response:

We thank the reviewer for this important comment. We agree that immunohistochemical detection of C3c and immunoglobulins does not directly demonstrate immune complex formation or definitive activation of the complement cascade. We did not perform direct immunofluorescence or additional staining for C4d or C5b-9 in the present study.

Accordingly, we have revised the manuscript to avoid overstatement. Specifically, we replaced expressions such as “complement activation” with “complement deposition” and modified the text to indicate that our findings are “consistent with” rather than confirmatory of immune complex-mediated injury.

Based on the reviewer's suggestions, we modified the manuscript as follows:

(Page 2, line 19) DM skin is characterized by the deposition of immunoglobulins and complement C3c at sites of interface dermatitis, findings that are consistent with immune complex-mediated injury.

(Page 10, line 13) These findings are consistent with a possible role of immune complex-mediated injury in the development of dermatitis in DM. Taken together, these findings suggest that skin lesions in dermatomyositis are best characterized as interface dermatitis accompanied by immunoglobulin and complement deposition, findings that are consistent with immune complex-mediated injury rather than nonspecific immunoglobulin accumulation.

(Page 11, line 11) Collectively, these findings suggest that immunoglobulin and complement deposition, together with inflammatory cell infiltration, may contribute to cutaneous inflammation in patients with DM.

(Page 13, line 1) Our findings revealed strong deposition of complement C3c, IgM, and IgA in interface dermatitis in DM, findings that are consistent with immune complex-mediated injury.

3. In dermatopathology, direct immunofluorescence performed on frozen tissue sections is the established method for evaluating immunoglobulin and complement deposition at the dermo-epidermal junction. The authors should explain their rationale for selecting formalin-fixed paraffin-embedded immunohistochemistry, describe how nonspecific serum protein trapping or vascular leakage was excluded, and specify the negative controls used for the rabbit primary antibodies. This concern is particularly relevant given that IgG staining was moderate to strong in both groups, which raises the possibility of background or nonspecific deposition.

Authors’ response:

We thank the reviewer for this important and insightful comment. We agree that direct immunofluorescence using frozen tissue sections is the standard method for evaluating immunoglobulin and complement deposition in dermatopathology. In the present study, however, we used formalin-fixed paraffin-embedded archival specimens because frozen tissue was not available for this retrospective analysis, and all specimens had been collected as part of routine clinical practice.

We have revised the Methods section to clarify the rationale for using immunohistochemistry with formalin-fixed paraffin-embedded tissues. We have also clarified that mouse IgG1 was used as a negative control for our in-house anti-human MDA5 monoclonal antibody (clone H27). We did not perform separate species-matched negative controls for the rabbit primary antibodies against C3c and immunoglobulins, and we acknowledge that this represents a methodological limitation.

In addition, we have expanded the Discussion to note that, because the present study relied on immunohistochemistry rather than direct immunofluorescence, and because species-matched negative controls were not performed for the rabbit primary antibodies, nonspecific trapping of serum proteins and vascular leakage cannot be completely excluded. We also added a brief discussion of the finding that IgG staining was observed in both dermatomyositis and control skin, consistent with previous reports describing baseline immunoglobulin deposition in normal human skin.

Accordingly, we have revised the manuscript as follows to clarify these methodological considerations and to more explicitly acknowledge the limitations of the immunohistochemical approach used in this study.

(Page 7, line 5) Direct immunofluorescence using frozen tissue sections is the standard method for evaluating immunoglobulin and complement deposition in dermatopathology. In the present study, we used formalin-fixed paraffin-embedded archival specimens because frozen tissue was not available for this retrospective analysis and all specimens had been collected as part of routine clinical practice.

(Page 7, line 20) mouse IgG1 (BioLegend Japan, Tokyo, Japan) was used as a negative control for the in-house anti-human MDA5 monoclonal antibody (clone H27). Separate species-matched negative controls were not performed for the rabbit primary antibodies against C3c and immunoglobulins.

(Page 13, line 3) Fourth, direct immunofluorescence, which is the standard method for evaluating immunoglobulin and complement deposition in dermatopathology, was not performed because frozen tissue was not available. In addition, species-matched negative controls were not separately performed for the rabbit primary antibodies. Therefore, nonspecific trapping of serum proteins and vascular leakage cannot be completely excluded.

4. Although a 0–3 ordinal scoring system was employed, the results are reported as mean ± SD, which is not appropriate for ordinal data; median and interquartile range should be reported instead. In addition, the manuscript should specify how many pathologists evaluated the slides, whether scoring was performed independently prior to reaching consensus, and whether interobserver agreement was formally assessed. Reporting the full distribution of scores for each marker, along with exact p-values, would improve transparency. The authors should also clarify whether any correction for multiple comparisons was considered.

Authors’ response:

We thank the reviewer for this important and constructive comment. We agree that, because the immunohistochemical scores were based on an ordinal 0–3 scale, reporting the results as mean ± standard deviation was not the most appropriate approach. Accordingly, we have revised Table 2 to present the data as median (interquartile range) while retaining the full distribution of scores (0/1/2/3) for each marker.

We have also expanded the Methods section to provide additional details regarding the pathological assessment procedure. Specifically, we now state the number of pathologists involved in the evaluation, clarify that all slides were reviewed while blinded to the clinical information, and describe that the final scores were determined by consensus after independent assessment.

In addition, we have revised Table 2 to report numerical p-values rather than threshold expressions whenever possible. Because five immunohistochemical markers were compared between the dermatomyositis and control groups, we additionally performed Bonferroni correction as a sensitivity analysis (adjusted significance threshold, p < 0.01). The significant differences observed for C3c, IgM, and IgA remained statistically significant after correction, confirming the robustness of these findings.

Formal interobserver agreement was not assessed in the present study. We agree that this is an important methodological consideration and have acknowledged in the Discussion that the semi-quantitative scoring system remains inherently subjective despite blinded evaluation and consensus review.

These revisions have been incorporated into the Methods, Results, Table 2, and Discussion sections of the manuscript as follows:

(Page 8, line 9) Three evaluators (YZ, MT, and JA) independently reviewed all slides while blinded to clinical information, and the final scores were determined by consensus.

(Page 8, line 14) Because five immunohistochemical markers were compared between groups, Bonferroni correction was additionally performed as a sensitivity analysis. A p value of <0.01 was considered statistically significant after correction.

(Page 13, line 13) Formal interobserver agreement was not assessed, and the semi-quantitative scoring system remains inherently subjective despite blinded evaluation and consensus review.

5. The manuscript indicates that the expanded cohort was assembled following the initial observations and that the two cohorts were subsequently combined. This approach requires clarification. If the expanded cohort was recruited after reviewing the exploratory results, pooling the data may introduce bias, and it should be stated explicitly whether this pooled analysis was prespecified. At a minimum, results from the two cohorts should be presented separately, even if a combined analysis is retained, in order to demonstrate consistency across cohorts.

Authors’ response:

We thank the reviewer for this insightful comment. We agree that the relationship between the exploratory study and the expanded cohort study required further clarification.

The expanded cohort study was conducted to confirm the observations obtained in the exploratory study using the same inclusion criteria, immunohistochemical procedures, semi-quantitative scoring system, and statistical methods. As originally planned, the final analysis was performed using the pooled dataset after confirming that the overall staining patterns and group differences were generally consistent between the two cohorts.

To improve transparency, we have revised the Methods section to clarify this study design. In addition, we have provided the results of the exploratory study and the expanded cohort study separately in Supporting Information (S1 Table), while retaining the pooled analysis as the primary analysis.

In the expanded cohort, MDA5 staining scores were significantly lower in control skin than in dermatomyositis skin. However, several control specimens in this cohort were older archival samples, and reduced staining intensity related to long-term storage may have contributed to this finding. We have added this point to the manuscript as follows:

(Page 6, line 12) The expanded cohort was assembled to confirm the observations obtained in the exploratory study using the same immunohistochemical and statistical methods. As originally planned, the final analysis was performed using the pooled dataset after confirming that the findings were directionally consistent between the two cohorts.

(Page 10, line 3) The results of the exploratory study and the expanded cohort study analyzed separately are provided in S1 Table. Overall staining patterns and group differences were generally consistent between the two cohorts. In the expanded cohort, MDA5 staining scores were significantly lower in control skin than in dermatomyositis skin; however, several control specimens were older archival samples, and reduced staining intensity related to long-term storage may have contributed to this finding.

6. The authors note that MDA5 staining appeared relatively weak in several older specimens, raising concern about pre-analytic variability more broadly. The manuscript should report, where not already provided, the distribution of specimen age, fixation conditions, anatomical biopsy site, disease duration, and treatment status at the time of biopsy. In particular, prior use of systemic corticosteroids or immunosuppressive therapy could influence both inflammatory infiltrates and protein deposition and should therefore be described and discussed.

Authors’ response:

We thank the reviewer for this important comment. We agree tha

Attachment Submitted filename: MDA5_Skin_R1_TH.docx

# Peer review: Decision Letter 1

**DOI:** 10.1371/journal.pone.0351248.r004

**Authors:** Seth Domfeh

25 May 2026

<p>Expression of Melanoma Differentiation–Associated Gene 5 in the Epidermis and Cutaneous Deposition of Complement C3 and Immunoglobulins in Patients with Dermatomyositis

PONE-D-26-04603R1

Dear Dr. Hoshino,

We’re pleased to inform you that your manuscript has been judged scientifically suitable for publication and will be formally accepted for publication once it meets all outstanding technical requirements.

Within one week, you’ll receive an e-mail detailing the required amendments. When these have been addressed, you’ll receive a formal acceptance letter and your manuscript will be scheduled for publication.

An invoice will be generated when your article is formally accepted. Please note, if your institution has a publishing partnership with PLOS and your article meets the relevant criteria, all or part of your publication costs will be covered. Please make sure your user information is up-to-date by logging into Editorial Manager at Editorial Manager® and clicking the ‘Update My Information' link at the top of the page. For questions related to billing, please contact billing support.

If your institution or institutions have a press office, please notify them about your upcoming paper to help maximize its impact. If they’ll be preparing press materials, please inform our press team as soon as possible -- no later than 48 hours after receiving the formal acceptance. Your manuscript will remain under strict press embargo until 2 pm Eastern Time on the date of publication. For more information, please contact onepress@plos.org.

Kind regards,

Seth Agyei Domfeh, PhD

Academic Editor

PLOS One

Additional Editor Comments (optional):

Reviewers' comments:

Reviewer's Responses to Questions

**Comments to the Author**

1. If the authors have adequately addressed your comments raised in a previous round of review and you feel that this manuscript is now acceptable for publication, you may indicate that here to bypass the “Comments to the Author” section, enter your conflict of interest statement in the “Confidential to Editor” section, and submit your "Accept" recommendation.

Reviewer #1: All comments have been addressed

Reviewer #2: All comments have been addressed

**********

2. Is the manuscript technically sound, and do the data support the conclusions?

The manuscript must describe a technically sound piece of scientific research with data that supports the conclusions. Experiments must have been conducted rigorously, with appropriate controls, replication, and sample sizes. The conclusions must be drawn appropriately based on the data presented.

Reviewer #1: Yes

Reviewer #2: Yes

**********

3. Has the statistical analysis been performed appropriately and rigorously?

Reviewer #1: Yes

Reviewer #2: Yes

**********

4. Have the authors made all data underlying the findings in their manuscript fully available?

The PLOS Data policy requires authors to make all data underlying the findings described in their manuscript fully available without restriction, with rare exception (please refer to the Data Availability Statement in the manuscript PDF file). The data should be provided as part of the manuscript or its supporting information, or deposited to a public repository. For example, in addition to summary statistics, the data points behind means, medians and variance measures should be available. If there are restrictions on publicly sharing data—e.g. participant privacy or use of data from a third party—those must be specified.

Reviewer #1: Yes

Reviewer #2: Yes

**********

5. Is the manuscript presented in an intelligible fashion and written in standard English?

PLOS ONE does not copyedit accepted manuscripts, so the language in submitted articles must be clear, correct, and unambiguous. Any typographical or grammatical errors should be corrected at revision, so please note any specific errors here.

Reviewer #1: Yes

Reviewer #2: Yes

**********

6. Review Comments to the Author

Please use the space provided to explain your answers to the questions above. You may also include additional comments for the author, including concerns about dual publication, research ethics, or publication ethics. (Please upload your review as an attachment if it exceeds 20,000 characters)

Reviewer #1: (No Response)

Reviewer #2: Thank you authors for this medications. I have no more doubts , all of my previous suggestions are addressed.

**********

7. PLOS authors have the option to publish the peer review history of their article (what does this mean?). If published, this will include your full peer review and any attached files.

If you choose “no”, your identity will remain anonymous but your review may still be made public.

**Do you want your identity to be public for this peer review?** For information about this choice, including consent withdrawal, please see our Privacy Policy.

Reviewer #1: No

Reviewer #2: **Yes:** Asaad Babker

**********

# Editor report: Acceptance letter

**DOI:** 10.1371/journal.pone.0351248.r005

**Authors:** Seth Domfeh

PONE-D-26-04603R1

PLOS One

Dear Dr. Hoshino,

I'm pleased to inform you that your manuscript has been deemed suitable for publication in PLOS One. Congratulations! Your manuscript is now being handed over to our production team.

At this stage, our production department will prepare your paper for publication. This includes ensuring the following:

* All references, tables, and figures are properly cited

* All relevant supporting information is included in the manuscript submission,

* There are no issues that prevent the paper from being properly typeset

You will receive further instructions from the production team, including instructions on how to review your proof when it is ready. Please keep in mind that we are working through a large volume of accepted articles, so please give us a few days to review your paper and let you know the next and final steps.

Lastly, if your institution or institutions have a press office, please let them know about your upcoming paper now to help maximize its impact. If they'll be preparing press materials, please inform our press team within the next 48 hours. Your manuscript will remain under strict press embargo until 2 pm Eastern Time on the date of publication. For more information, please contact onepress@plos.org.

You will receive an invoice from PLOS for your publication fee after your manuscript has reached the completed accept phase. If you receive an email requesting payment before acceptance or for any other service, this may be a phishing scheme. Learn how to identify phishing emails and protect your accounts at https://explore.plos.org/phishing.

If we can help with anything else, please email us at customercare@plos.org.

Thank you for submitting your work to PLOS ONE and supporting open access.

Kind regards,

PLOS ONE Editorial Office Staff

on behalf of

Dr. Seth Agyei Domfeh

Academic Editor

PLOS One
